# The impact of exercise on protein levels in *Drosophila melanogaster*

**DOI:** 10.1242/bio.062342

**Published:** 2026-01-29

**Authors:** James C. Walts, Ami A. Dave, Nicole C. Riddle

**Affiliations:** Department of Biology, University of Alabama at Birmingham, Birmingham, AL, 35294, USA

**Keywords:** *Drosophila*, Exercise physiology, Metabolites, TreadWheel, GWAS

## Abstract

Moderate exercise is recommended by health experts across the globe to maintain health. Exercise induces a range of physiological changes, often shifting body composition towards increased muscle mass. To investigate the genetic factors controlling exercise responses, particularly altered body composition, we measured protein levels, as a proxy for muscle mass, in 32 genetically distinct strains from the *Drosophila melanogaster* Genetic Reference Panel (DGRP) that underwent a 5-day exercise treatment. At baseline, the protein levels varied significantly across genotypes and between sexes. The effects of exercise on protein content also were highly variable: some strains showed increased levels, others decreased levels, and many strains showed no significant change. A genome-wide association study (GWAS) identified multiple loci linked to both baseline and exercise-induced protein levels, as well as the change in protein levels after exercise. Many of these loci are involved in morphogenesis, neuronal development, and cell signaling. Notably, there was no correlation between protein concentration and measures of activity levels or climbing speed, suggesting muscle mass and function may be regulated independently. A modest positive correlation between protein levels and lifespan was observed in exercise-treated females, but not in other groups. These findings highlight the complex, context-dependent genetic architecture underlying exercise responses and underscore the need to consider both genotype and sex in physiological exercise studies. The genes identified here provide targets for future work aimed at elucidating the molecular mechanisms of exercise response.

## Teaser

**Summary:** Whole-body protein levels respond to exercise in a sex- and genotype-dependent manner, with candidate genes linked to neuronal differentiation and development contributing to this variability in response.

## INTRODUCTION

Adequate levels of physical activity are essential for a healthy life (Bize et al., 2007; Qiu et al., 2023; Ruegsegger and Booth, 2018). Due to the sedentary nature of many occupations, exercise is recommended by public health organizations to achieve health-promoting activity levels. For example, the Center for Disease Control (CDC) recommends that adults aim for 30 min of moderate intensity exercise per day (Physical Activity Guidelines Advisory Committee, 2018; U.S. Department of Health and Human Services, 2018). Achieving these minimal activity levels has a range of health benefits, including a reduced risk of obesity, diabetes, cardiovascular disease, cancer, and more (Arya et al., 2010; Martinez-Vizcaino et al., 2024; Posadzki et al., 2020; Qiu et al., 2023; Reimers et al., 2012; https://progressreport.cancer.gov/). In addition, the recommended exercise levels are chosen to promote healthy aging, decreasing muscle loss with age, maintaining mobility, and reducing the risk for frailty and falls (Physical Activity Guidelines Advisory Committee, 2018; Eckstrom et al., 2020; Qiu et al., 2023; Yang et al., 2024). These diverse benefits illustrate why public health organizations emphasize the inclusion of exercise into daily life and actively promote for processes and structures that encourage exercise.

Despite the important health benefits of exercise, less than half of all adults in the United States meet the CDC recommendations for exercise (2024; Carlson et al., 2010). While this number includes individuals that choose not to exercise, there are also significant numbers of individuals that cannot exercise for a variety of reasons, including age, physical limitations, and disease. Thus, there is a growing interest in the development of exercise mimetics – procedures or drugs that can mimic the health-promoting benefits of exercise without requiring physical exercise (Crunkhorn, 2022; Fan and Evans, 2017; Hawley et al., 2021). Currently, many of the molecular events precipitated by exercise, particularly those pathways mediating the long-term effects, are poorly understood and in need of further study (but see Chen et al., 2022; Eissenberg, 2022; MoTrPAC Study Group et al., 2024). To develop exercise mimetics – and optimized exercise programs – it is essential that we understand the molecular processes that lead to the health-promoting effects of exercise. Exercise mimetics need to be able to activate the same molecular pathways as exercise to achieve similar outcomes and thus expand the health-promoting effects of exercise to the population of individuals that do not meet physical activity guidelines.

One primary target of exercise is muscle tissue. During exercise, muscle tissues utilize a large amount of energy, mobilized from both intramuscular energy stores, as well as provided by extramuscular energy stores through the bloodstream (Coyle, 1995; Hargreaves and Spriet, 2020; Murach and Bagley, 2025). Exercise also causes damage to muscle, which stimulates repair and regeneration of muscle (Chen et al., 2022; Hawley et al., 2014; Qiu et al., 2023). Frequent exercise leads to an increase in muscle mass and heightened repair/regenerative function of the muscle tissue (Cartee et al., 2016; Chen et al., 2022). Through these processes, exercise counteracts and prevents the muscle loss typically associated with aging (Qiu et al., 2023). Thus, muscle responds to exercise with a variety of physiological changes, including changes in metabolite levels and muscle mass.

*Drosophila melanogaster* is a well-known genetic model system that also serves as a model for exercise research (Bilder and Irvine, 2017; Riddle, 2019; Sujkowski and Wessells, 2018; Verheyen, 2022; Watanabe and Riddle, 2019). Several systems exist that allow experimenters to induce physical activity in the animals, forcing them to exercise beyond their preferred baseline level of activity (Mendez et al., 2016; Piazza et al., 2009; Watanabe and Riddle, 2019). The Treadwheel exploits the animals' innate negative geotaxis (the tendency to seek out the top of their enclosure) and uses slow rotation of the fly enclosures to exercise the animals (Mendez et al., 2016). Treatments on the Treadwheel increase physical activity levels above baseline, changing metabolite levels, expression levels of mitochondrial genes, climbing speed, and weight (Mendez et al., 2016; Riddle, 2020; Watanabe et al., 2020; Watanabe and Riddle, 2017, 2021a,b). Like in humans, the effect of exercise in *D. melanogaster* are highly genotype- and sex-dependent, making *Drosophila* an excellent model for exercise research.

Here, we use *D. melanogaster* to investigate how protein levels change in response to an exercise treatment. We used strains from the *D. melanogaster* Genetics Reference Panel (DGRP), a wild-derived, fully genotyped strain collection that is suitable for genome-wide association study (GWAS) (Huang et al., 2014; Mackay et al., 2012). We used an exercise treatment that we have shown to induce increased activity levels in animals from the DGRP strains (Watanabe et al., 2020). The animals used in this study exhibit changes in body weight and climbing ability with exercise in a sex- and genotype-dependent way, findings previously published by our group (Riddle, 2020; Watanabe et al., 2020; Watanabe and Riddle, 2021a,b). Measuring protein levels in both control and exercise-treated animals, we wanted to gain insights into the mechanisms mediating the exercise-induced changes in body mass and climbing ability and identify candidate genes that control the level of response observed. Using protein levels as a proxy for muscle mass, we found that protein levels strongly depend on sex and genotype. A GWAS suggests that genes involved in morphogenesis, in particular in the central nervous system, contribute to the variation in protein levels in these strains. The response in protein levels to exercise among the strains investigated is diverse, including strains with increased and decreased protein levels, as well as strains that show no change. A GWAS links this change in protein levels to genes involved in the response to signals and morphogenesis. Together, these findings highlight the importance of studying a range of genotypes and both sexes and the challenges of identifying broadly generalizable principles regarding exercise that apply across diverse individuals.

## RESULTS

### Protein levels vary significantly among DGRP strains, both with and without exercise-treatment

As exercise has the potential to cause a shift in body composition, reducing fat mass and increasing muscle mass (for example, see Christiansen et al., 2010), this shift might be detected by increased, whole body protein levels. Thus, we measured protein levels in individuals that received a 5-day exercise treatment (Watanabe and Riddle, 2021a,b), which increases the activity level on average six-fold during exercise with 80% of males and 90% of female groups showing an increase (Watanabe et al., 2020). We compared protein levels from exercised animals to matched control samples that did not receive an exercise treatment. We carried out two different analyses, first examining protein amount per animal. This measure is impacted by body size, and females typically are larger than males. Thus, we also examined protein amounts per mg animal weight, which removes effects of body size and reveal insights about body composition. The analyses in the main figures focuses on the ‘per fly’ data, while the ‘per weight’ data are presented in the supplementary information.

**Fig. 1.**
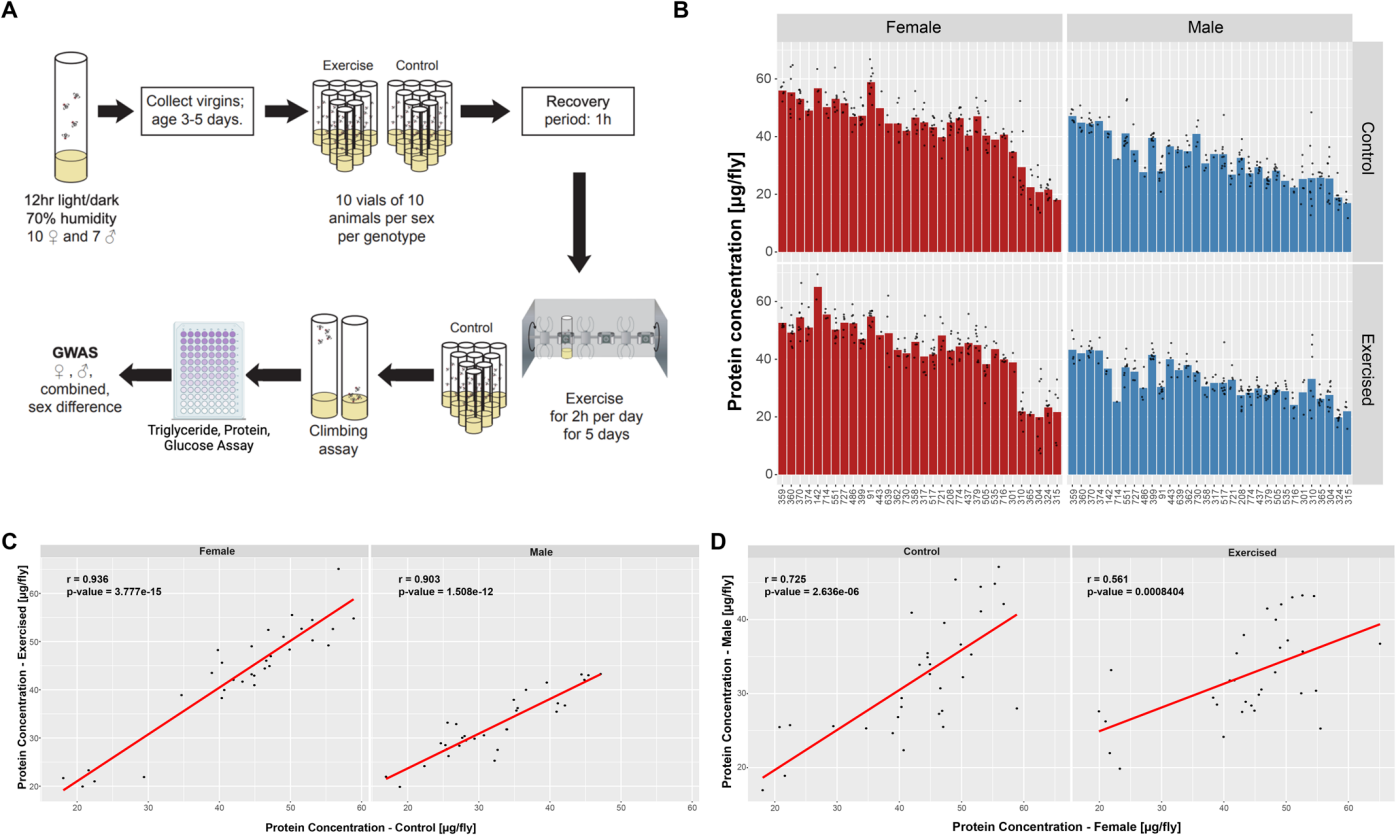
**Protein levels significantly vary among DGRP lines normalized by fly number.** (A) Experimental set-up. 3 to 5-day-old virgin flies were collected and separated into groups of ten by sex, using ten vials of ten flies per sex/genotype/treatment combination. Flies in the exercise-treated group underwent five consecutive days of exercise using the Treadwheel, while animals in the control group were handled the same way, but without the treatment on the Treadwheel. 1 day after completing the exercise treatment, climbing ability of all animals was measured. At this point, the animals were weighed and frozen for metabolite analysis. (B) Protein levels per fly are shown for control (C, top) and exercise-treated (T, bottom) animals separated by sex with females on the left in red and males on the right in blue. X-axis: DGRP line number; Y-axis: protein amount per fly in µg/fly. Group means are shown as bars, with individual data points plotted. *n*=1-10 per group, with ten flies per vial. Each data point represents the mean from three technical replicates averaged per vial. (C) Mean protein amounts per fly (in µg/fly) of control (Y-axis) and exercise-treated (X-axis) animals are plotted separately against each other for females (left; r=0.936, *P*-value=3.777e-15) and males (right; r=0.903, *P*-value=1.508e-12). *n*=1-10 biological replicates (vials) per genotype, with ten flies per vial. Each data point represents the mean from three technical replicates averaged per vial. *P*-values and Pearson's correlation coefficient (r) are included within each graph. The trendline is shown in red. (D) Mean protein amounts per fly (in µg/fly) of females (X-axis) are plotted against measurements from males (Y-axis) separately for control (left; r=0.725, *P*-value=2.636e-0.6) and exercise-treated (right; r=0.561, *P*-value=0.0008404) animals of each DGRP strain. *n*=1-10 biological replicates (vials) per genotype, with ten flies per vial. Each data point represents the mean from three technical replicates averaged per vial. *P*-values and Pearson's correlation coefficient (r) are included within each graph. The trendline is shown in red.

In both analyses, we found extensive variation in protein levels among the 32 DGRP strains included in this study. Examining total protein per fly, the overall highest protein level was 65.1±6.2 µg/fly and the overall lowest protein level was 16.9±3.8 µg/fly (Fig. 1B), revealing an approximate four-fold difference between animals of the highest and lowest protein amounts. Amongst females, the highest protein amounts were recorded for line 91 in control animals and for line 142 among treated animals. The lowest protein amounts for females were recorded for control animals of line 304 and treated animals of line 315. The genotypes with the highest or lowest protein amounts are different in males; line 359 had the highest protein amount in both the control and treatment groups. Males of line 315 had the lowest protein amount in the control group and line 324 in the exercise-treated samples. Given these data, protein amount per fly varied between the experimental groups included in this study.

While there was significant variation in protein amounts among all groups, females overall had higher protein amounts than males (*P*<2.2e-16, Kruskal–Wallis rank sum test). This larger amount of protein is likely due to females' overall larger size (*P*=≤2.2e-16, Kruskal–Wallis rank sum test; for the weights of these animals, see Watanabe and Riddle, 2021b). If the body composition is identical between the sexes, we would expect to detect more protein in female samples. To check if there was any indication that the body composition might differ between the sexes, we also analyzed protein levels per sample weight. We found that overall, protein levels range from a low of 16.4±2.9 µg/mg (exercise-treated females, line 310) to a high of 70.4±3.9 µg/mg (control males, line 359), representing a four-fold difference between the animals with the highest and lowest protein amounts per weight (Fig. S1A). Protein amounts were highest for females of line 359 for the control and 639 for the exercise treatment, and lowest for females of line 315 for the control and 310 for the exercise treatment. Among males, line 359 had the highest protein amounts among the control and the exercise-treated animals. Line 315 males showed the lowest protein amounts among the control and exercise-treated animals. Overall, irrespective of the measurement unit, these data demonstrate that our study population showed a significant degree of variation in protein levels, both under control conditions and with exercise treatment.

### Sex and genotype strongly impact protein levels

Next, we investigated how protein levels are affected by sex and treatment conditions. We plotted mean protein amount of females against measurements from males of the same DGRP line separated by treatment groups (Fig. 1D and Fig. S1C). We found a strong positive correlation between protein amount of females and males of the same genotype in both treatment groups (control group: Pearson's correlation coefficient r=0.725, *P*=2.636e-06; exercise group: Pearson's correlation coefficient r=0.561, *P*=0.0008404; Fig. 1D). Examining how protein amount differed between the treatment groups, we found that for females, there was a strong positive correlation (Pearson's correlation coefficient r=0.936, *P*=3.777e-15) between control and exercise-treated animals. Similarly, male control and exercise-treated animals also showed a strong positive correlation (Pearson's correlation coefficient r=0.903, *P*=1.508e-12; Fig. 1C). We found similarly strong positive correlations between protein per weight of females and males in both treatment groups (control group: Pearson's correlation coefficient r=0.716, *P*=4.145e-06; exercise group: Pearson's correlation coefficient r=0.504, *P*=0.003269; Fig. S1C), and between treatment groups for each sex (females: r=0.906, *P*=9.702e-13; males: r=0.842, *P*=1.539e-09; Fig. S1B). These results suggest a strong effect of genotype on protein amount (per fly and per weight), irrespective of sex or treatment. Given that the correlation between treatments within each sex was stronger than the correlation between sex, within treatment correlation (Fisher's z transformation, *P*<0.05), these data also raise the possibility that the sex of the animal might impact protein levels more strongly than the treatment condition.

To gain further insights into how the observed variation in protein levels arises, we explicitly tested for the impact of sex, genotype, and treatment. As the protein levels were not normally distributed (Shapiro-Wilk normality test, protein per fly, *P*=9.694e-06; protein per mg body weight, *P*=5.63e-11), we used non-parametric tests. We found that both genotype and sex strongly impacted protein levels (Kruskal–Wallis test, protein per fly, *P*<2.2e-16 and *P*<2.2e-16; protein per mg body weight, *P*<2.2e-16 and *P*=7.827e-06), while treatment showed no overall effect across all strains (protein per fly *P*=0.7766; protein per mg body weight *P*=0.7995). Because our previous work on exercise-related traits has demonstrated the importance of interaction effects, we used generalized linear models to investigate these effects. For both the protein per fly data and the protein per mg body weight data, we found that the genotype by sex interaction impacted protein levels (13 of 31 comparisons are significant with *P*<0.05 in the per fly analysis, 7 of 31 in the per weight analysis; Table S3). Thus, animal protein levels depend on the interactions between treatment, sex, and genotype.

### GWAS identifies genes that control protein levels in control and exercise-treated animals

We next wanted to identify the genes that control protein levels in *D*. *melanogaster*. Thus, to confirm if a GWAS would be appropriate to investigate protein amounts, a range of genetic parameters were calculated, including heritability (Table S4). Heritability (H_2_) for protein levels was high, for both per-fly (control animals: 0.791; treated animals: 0.788) and per-weight (control animals: 0.722; treated animals: 0.673) data. These findings confirm that a GWAS analysis is appropriate to investigate the genetic basis of variation in protein levels in the DGRP.

To identify genetic variants linked to protein levels, we used a mixed model that accounts for common inversions present in the DGRP as well as Wolbachia infection status in addition to sex and genotype (implemented through the DGRP2 webtool). Four separate analyses were carried out for both per-fly and per-weight protein amounts to identify genetic variants linked to protein amounts in females, males, overall (combined data from males and females), and to the difference in protein amounts between females and males (Tables S5, S6). We found that in the control animals, 72 genetic variants are identified linked to per-fly protein amounts (Table 1), 37 variants coming from the analysis of females, 26 variants from the analysis of males, 16 variants from the combined sex analysis, and no variants are linked to the difference between the two sexes. In the analysis of per-fly protein amounts from the exercise-treated animals, we identified a similar number of genetic variants (74 total, 34 for female, 19 for male, 36 for combined sex data, and two for the sex difference; Table 1). Carrying out the same analysis on the per-weight protein amounts identifies a similar number of genetic variants with 64 variants linked to protein amounts in the control animals (20 for female, 15 for male, 22 for combined sex data, and 15 for the sex difference; Table 1) and 42 variants in the exercise-treated animals (11 for female, 12 for male, seven for combined sex data, and 11 for the sex difference; Table 1). Together, these analyses demonstrate that dozens of genetic loci contribute to the control of protein amount in *Drosophila*. Furthermore, they suggest that the sex differences seen are genetic in origin.

**Table 1.** GWAS results. The number of genetic variants linked to protein levels in control animals, exercise-treated animals, or the change in protein levels seen with exercise are shown

Phenotype	Number of variants				
	Female	Male	Combined	Difference between sexes	Total
Protein amount per fly					
Control animals	37	26	16	0	72
Treated animals	34	19	36	2	74
Change	9	10	29	0	50
Protein amount per weight					
Control animals	20	15	22	15	64
Treated animals	11	12	7	11	42
Change	30	4	4	7	68

Number of genetic variants linked to protein levels in the various GWASs are shown. Female, number of variants detected using the data from females only. Male, number of variants identified using the data from males only. Combined, number of variants identified using the combined data from both sexes. Difference between sexes, number of variants identified as linked to the difference in phenotype between the sexes. Total, total number of variants identified for a given phenotype in a specific dataset. Top: results for protein amount per fly; bottom: results for protein amount per weight.

**Fig. 2.**
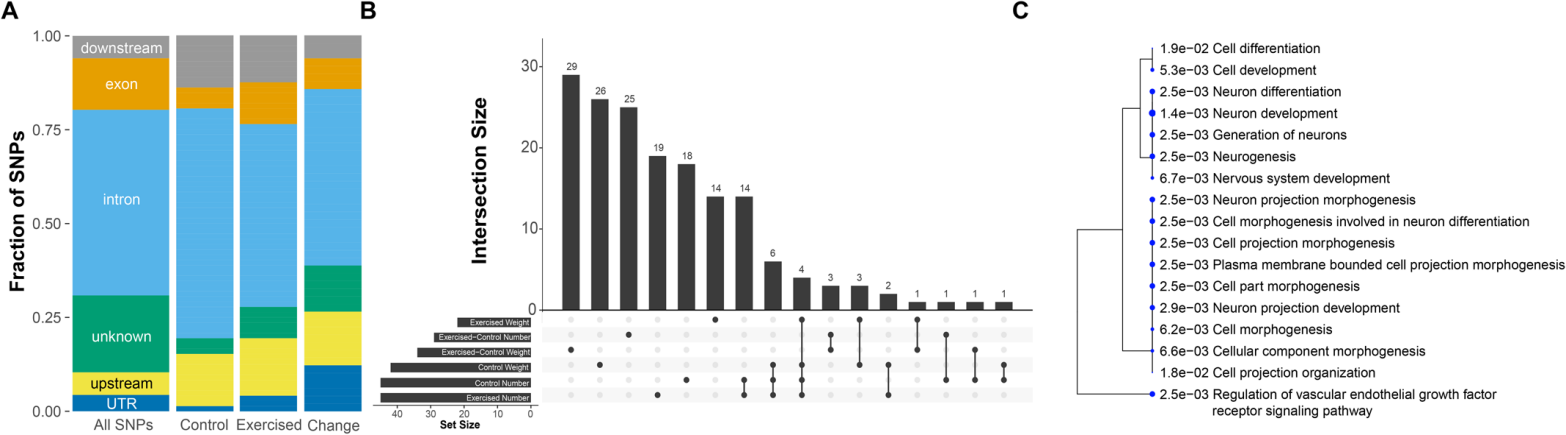
**GWAS identifies a diverse set of candidate genes as linked to protein levels in both control and exercise-treated animals.** (A) The genetic variants included in the GWAS analysis are classified based on their location relative to genes. The fraction of each class is shown in the stacked bar graph for the total set of variants included in the GWAS (left, ‘All SNPs’), for the variants identified as significant in the analysis of the ‘per fly’ data from control animals (‘Control’), and for the variants identified as significant in the analysis of the ‘per fly’ data from exercise-treated animals (‘Exercised’), as well as for the variants associated with the exercise-induced change in protein levels (‘Change’). The distributions are significantly different from each other (chi-square test, *P*<0.005497). (B) The overlap between the set of candidate genetic variants in the different GWASs are shown in this plot, with the size of the overlap (intersection size) shown in the bar graph above the diagram illustrating which groups are being compared. Number, analysis of protein amount per fly; weight, analysis of protein amount per weight. (C) Tree plot illustrating the relationship of the biological process GO terms identified as overrepresented among the candidate variant set shared between the ‘per fly’ control and exercise results. *P*-values are shown besides each GO term, and the size of the circles reflects the size of the gene set associated with the term.

The genetic variants linked to protein amount are distributed across the *Drosophila* genome and represent a range of locations with respect to genes. The variants included in the GWAS can be classified based on their association with annotated genes, either as ‘upstream’, ‘downstream’, ‘UTR’, ‘exon’, ‘intron’, or unknown, and genome-wide, the largest group of variants are within introns (Fig. 2A, left column). When the genetic variants detected as linked to per-fly protein amount are classified in this way, the fraction of variants in these six classes are significantly different from the genome-wide distribution, either for the analysis of the data from control animals or for the exercise-treated animals (chi-square test, *P*<0.005497; Fig. 2A). Carrying out the same analysis for the per-weight protein amounts reveals that the genetic variants identified differ in their classification from the genome-wide pattern for the control samples, but not the exercise-treated samples (chi-square test, control: *P*=0.02599 and exercise-treated: *P*=0.1584, Fig. S2). Thus, the genetic variants detected as impacting protein amount often are linked to genes, but not always more so than the total variant population investigated.

The genes identified in the GWAS as linked to protein levels in *D. melanogaster* are functionally diverse. Overall, there are 24 genes that are detected as linked to per-fly protein amount in both control and exercise-treated animals [Fig. 2B; *Apoltp*, *CG13531*, *CG17648*, *myd (CG31475)*, *CG32373*, *Mnr (CG32521)*, *CG33144*, *CG42747*, *CG4502*, *Spar (CG4577)*, *CG5835*, *CG9902*, *lncRNA:CR44632*, *Ca-alpha1D*, *NimC2*, *Pdp1*, *Ptp10D*, *Ptp61F*, *RpL23*, *cv-c*, *nompA*, *qin*, *sif*, *toc*]. This overlap is significantly higher than expected by chance (*P*=3.86222e-52; hypergeometric test), suggesting that this overlapping gene set can be considered as strong candidate genes. The results of the Gene Ontology (GO) term analysis for this gene set are illustrated in Fig. 2C (Table S7). Biological processes related to morphogenesis, particularly in the central nervous system, are over-represented, an enrichment that is due to a set of eight genes that are linked to neuronal development and differentiation.

Carrying out the same analysis with the per-weight protein amount, we identify 64 genetic variants linked to protein amount in the control animals and 42 variants in exercise-treated animals (Table 1). As in the per-fly data, these total counts include variants linked to protein amounts in males, females, both sexes, and some linked to the difference between the sexes (Table 1). There are variants in seven genes linked to protein amounts in both control and exercise-treated animals [Fig. 2B: *CG33144*, *Spar (CG4577)*, *lncRNA:CR43493*, *NimC2*, *Prp19*, *Ptp10D*, *SmydA-9*]. Again, this overlap between the two gene sets is significantly higher than expected by chance (*P*=3.980462e-10, hypergeometric test). No specific functional categories are overrepresented among these seven genes, which is not surprising given the small number of genes. There are four genes identified as candidate genes in the GWAS from the per-fly and the per-weight data (see Fig. 2B): *CG33144*, *Spar (CG4577)*, *NimC2*, and *Ptp10D*. We did not expect a lot of overlap between these gene sets, because reporting protein amount by fly number, the size of the animal plays an important role, while when reporting protein amount by fly weight, the body composition of the animal impacts the protein amount measured. Thus, the results from our GWAS illustrate that body size and body composition are controlled generally by different types of genes, with both gene sets impacting animal protein amounts with and without exercise.

### Protein levels show no correlation with animal activity levels or climbing speed

Because protein amounts provide an assessment of the animals' muscle mass, we asked how protein amount correlated with other exercise-related traits we measured in this strain collection previously (Watanabe et al., 2020; Watanabe and Riddle, 2021a,b). Specifically, we looked at the association of protein amount with animal activity (basal and exercise-induced activity; measured on a different cohort of animals from the same strains) and with climbing speed (measured on the same animals assayed here). When per-fly protein amounts were plotted against basal activity levels, no significant correlation was detected in either females or males of both treatment groups (Table 2 and Fig. S3; female control: r=−0.013, *P*-value=0.9505; female exercise-treated: r=−0.000, *P*-value=0.9984; male control: r=0.232, *P*-value=0.2744; male exercise-treated: r=0.244, *P*-value=0.2502). Likewise, there was no significant correlation between per fly protein amount and exercise-induced activity (Table 2 and Fig. S3; female control: r=−0.059, *P*-value=0.7839; female exercise-treated: r=−0.084, *P*-value=0.6974; male control r=0.133, *P*-value=0.5357; male exercise-treated r=0.178, *P*-value=0.4065). Analysis of the per-weight protein amounts showed a similar lack of correlation with activity levels (Table S8 and Fig. S3). Thus, the results from these correlation analyses suggest that there is no simple association between protein amount and animal activity.

**Table 2.** Correlations between protein levels and other exercise-related traits

Sex	Phenotype 1	Phenotype 2	Correlation coefficient (r^2^) (*P*-value)
Female	Protein concentration – control	Activity levels – basal	0.013 (0.9505)
Male	Protein concentration – control	Activity levels – basal	0.234 (0.2744)
Female	Protein concentration – exercise-treated	Activity levels – basal	0.000 (0.9984)
Male	Protein concentration – exercise-treated	Activity levels – basal	0.244 (0.2502)
Female	Protein concentration – control	Activity levels – exercise-induced	−0.059 (0.7839)
Male	Protein concentration – control	Activity levels – exercise-induced	0.133 (0.5357)
Female	Protein concentration – exercise-treated	Activity levels – exercise-induced	−0.084 (0.6974)
Male	Protein concentration – exercise-treated	Activity levels – exercise-induced	0.178 (0.4065)
Female	Protein concentration – control	Climbing speed – control	−0.229 (0.2316)
Male	Protein concentration – control	Climbing speed – control	−0.347 (0.07017)
Female	Protein concentration – exercise-treated	Climbing speed – exercise-treated	−0.181 (0.3465)
Male	Protein concentration – exercise-treated	Climbing speed – exercise-treated	−0.320 (0.09095)

Correlation analysis from protein amount per fly, showing Pearson's correlation coefficients as well as *P*-values.

While our analyses detected no correlation between protein amount and animal activity, it is possible that a more challenging physical activity might reveal additional insights. Thus, we investigated the relationship between protein amount and climbing speed (Watanabe and Riddle, 2021a). In females, there were no significant correlations in both control and exercise-treated animals between protein amount and climbing speed (Table 2, Table S8, and Fig. S4; per-fly data: control, r=−0.229, *P*-value=0.2316; treatment, r=−0.181, *P*-value=0.3465; per-weight data: control, r=−0.295, *P*=0.1205; treated, r=−0.252, *P*=0.187). Like females, males showed negative correlations between protein amount and climbing speed, but the association was not significant after adjusting for multiple testing (Table 2, Table S8, and Fig. S4; per-fly data: control, r=−0.347, *P*-value=0.07017; treatment, r=−0.320, *P*-value=0.09095; per-weight data: control, r=−0.377, *P*=0.04773; treated, r=−0.310, *P*=0.1012). Thus, animals with higher protein amount tended to have lower climbing speeds, but this association was not significant.

### The relationship between protein levels and lifespan is context-dependent

**Fig. 3.**
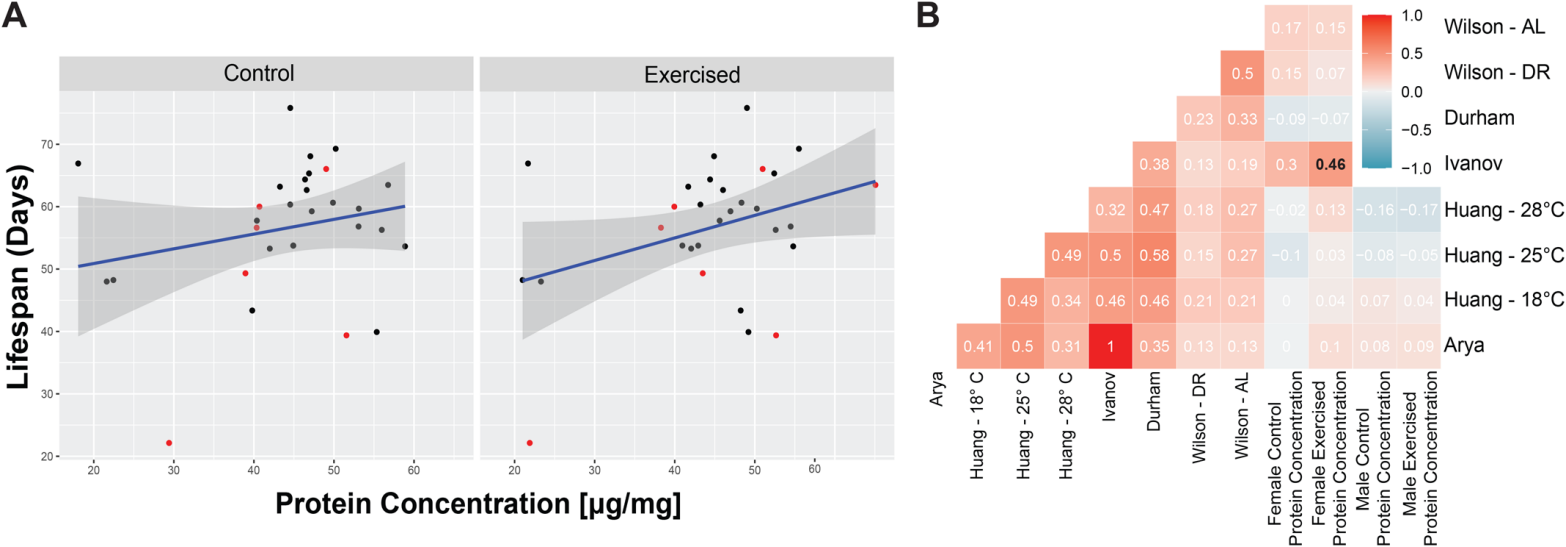
**Protein amount is moderately correlated to life span in females under specific conditions.** (A) Lifespan data from females are from (Ivanov et al., 2015). Mean protein amount per fly in µg/fly (X-axis), is plotted against lifespan in days (Y-axis) for control females (left) and exercise-treated females (right) from each genotype. The trendline is shown in blue. Additional data points included in the Ivanov et al. dataset but excluded from the Arya et al. dataset are shown in red (Arya et al., 2010; Ivanov et al., 2015). (B) Correlation matrix showing the Pearson correlation coefficients (r; using pairwise complete observations) between lifespan and protein levels (last four columns) as well as among the lifespan datasets.

As exercise, activity levels, and muscle mass also have the potential to impact the health- and lifespan of an individual, we investigated a potential link between protein amount and lifespan. As they matched our experimental design most closely, we used lifespan data from virgin females from Ivanov and colleagues for this analysis (Ivanov et al., 2015). There was a positive correlation between per-fly protein amounts and lifespan in both control and exercise-treated animals, but it was only significant in the exercise-treated animals (control, r=0.3, *P*=0.099; exercised, r=0.46, *P*=0.0098, Fig. 3). Similarly, for per-weight protein amounts, the correlation with lifespan does not meet the significance threshold in the control animals (r=0.26, *P*=0.2011, Fig. S5), but in exercise-treated animals, the correlation between lifespan and protein amount was significant (r=043, *P*=0.0281, Fig. S5). Thus, there is some indication that animals with higher protein amounts, that we expect to have more muscle mass, live longer than animals with a low protein amount, but in our study this relationship only holds for exercise-treated animals.

As the lifespan data from Ivanov and colleagues only includes female data, we sought out other lifespan datasets available for the DRGP strains that might be suitable to probe the relationship between protein amount and lifespan in both sexes. We identified four additional datasets, two from the Mackay lab that generated the DGRP strains (both sexes; Arya et al., 2010; Huang et al., 2020), one from the Kapahi lab (females only; Jin et al., 2020), and one from the Leips lab (females only; Durham et al., 2014). These studies differ significantly in the experimental conditions, with Arya and colleagues using virgin animals (the Ivanov study is a subset of the Arya et al. study published earlier) and all others using mated animals. In addition, the study by Huang and colleagues included three rearing temperatures (18°C, 25°C, 28°C; Huang et al., 2020), and the study by Jin and colleagues included two diets (*ad lib* and dietary restriction; Jin et al., 2020). While calculating the correlation between protein amount measured in our study with the lifespans measured in these four studies has some limitations due to differences in rearing conditions and diet, we were surprised to see that the correlation between protein amount and lifespan detected with the data from Ivanov was unique and could not be detected with any other dataset (Fig. 3B; r ranges from −0.17 to 0.17, all *P*-values >0.384 for the per-fly control dataset). This finding was particularly surprising for the dataset from Arya and colleagues (r=0.1, *P*=0.637 for the per-fly exercise-treated female dataset), as the Ivanov dataset represents a subset of this dataset published earlier. Further investigation revealed that (a) the correlation coefficients between lifespan measures from different studies tended to be relatively low (Fig. 3B), and (b) that the positive correlation detected with the lifespan data from Ivanov and colleagues is impacted by line 310, which has a low protein level and short lifespan, and it is not part of the Anya et al. dataset (the lines not included in Anya et al. are marked in red in Fig. 3). Together, these analyses suggest that there is an association between protein amount and lifespan across genotypes, but that the strength of this association is impacted strongly by the specific environmental conditions and genotypes. Given that the positive correlation was observed with lifespan measured under conditions most similar to our study, it is likely that lifespan and protein concentration change in concert under different experimental conditions. In addition, this finding indicates that the higher muscle mass suggested by high protein amount might be important for healthy aging and longer life.

### Genetics pathways involved in neuronal morphogenesis and cell-cell communication are linked to the exercise-induced change in protein levels

**Fig. 4.**
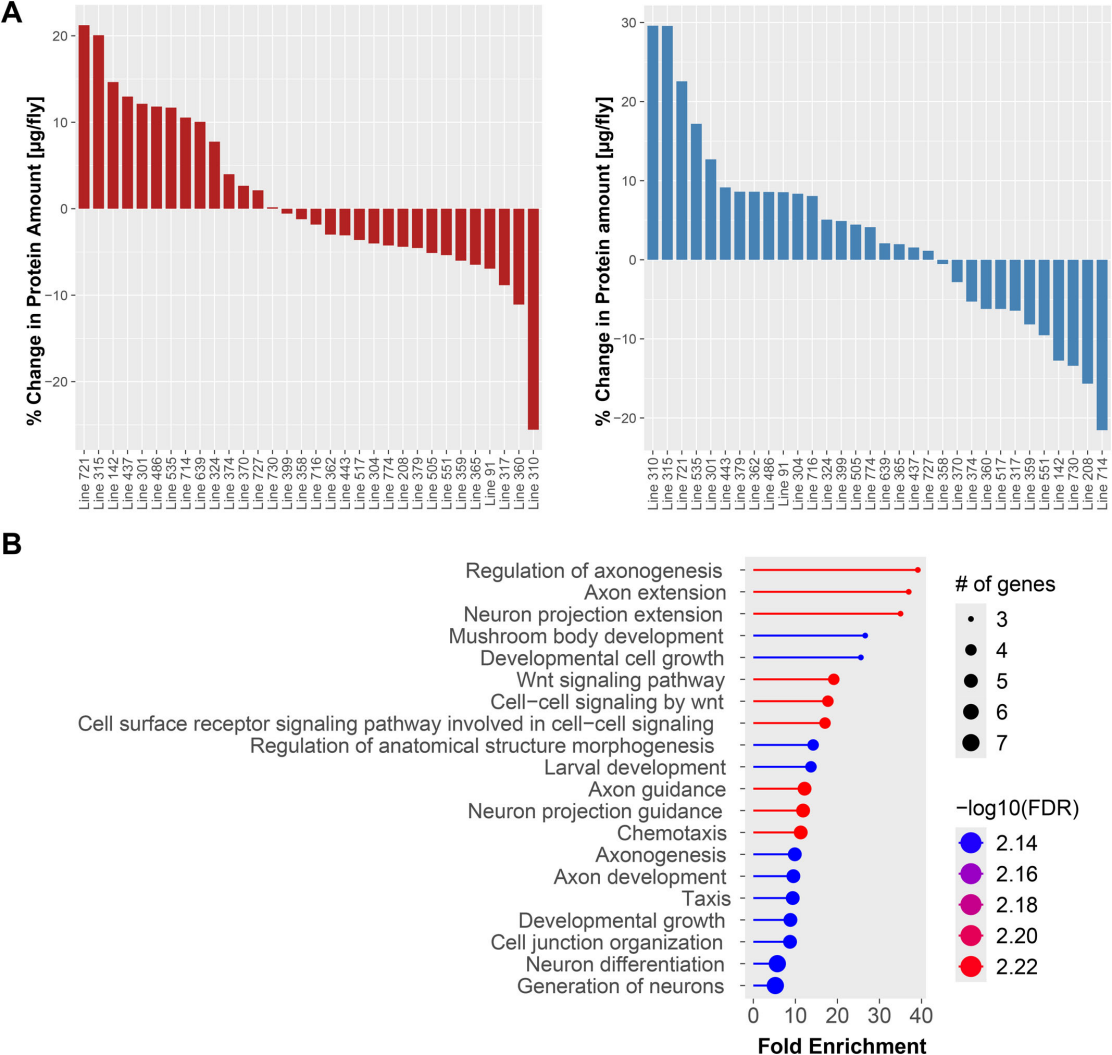
**Change in whole-body protein with exercise is linked to genes controlling morphogenesis and neuronal development.** (A) The change in protein with exercise (in percent, amount of protein per fly) is shown for females (left) and males (right). X-axis: DGRP line number; Y-axis: change in protein amount per fly in %. *n*=1-10 biological replicates (vials) per genotype, with ten flies per vial. (B) Biological process GO terms overrepresented among the candidate gene set identified as linked to the exercise-induced change in protein level by GWAS of data from protein amounts per fly. The level of overrepresentation is shown as fold enrichment within the graph, while the number of genes within the group is shown by the size of the circle. The color indicates the level of significance for the overrepresentation test.

Next, we investigated how protein amount changes with exercise. Typically, it is thought that exercise increases muscle mass, which could be measured by an increase in overall protein amount in the animal. Comparing the per-fly protein amount in animals of the same sex and genotype with and without an exercise treatment, we found that in females, protein amount change ranged from a 21% increase (line 721) to a 26% decrease (line 310), while in males the protein amount change ranged from a 30% increase (line 310) to a 22% decrease (line 714; Fig. 4A). The changes in protein are within a similar range in the per-weight data, +22% to −27% in females (lines 315 and 310) and +37% to −16% in males (lines 310 and 208; Fig. S6). These data illustrate that exercise treatments can lead to significant changes in protein amounts in these animals, but that the magnitude of this change is highly dependent on the sex and genotype of the animal.

To gain further insights into how protein amount changes in concert with other traits in response to exercise, we investigated possible links to change in activity level, climbing speed, and body weight with exercise that our lab previously measured (Watanabe et al., 2020; Watanabe and Riddle, 2021a,b) (Fig. S7). While we found no correlation between a change in climbing speed and a change in protein amount with exercise in females, in males, there was a negative correlation between these two traits (r=−0.39, *P*=0.04 for per-fly data, r=−0.32, *P*=0.09 for per-weight data). Thus, it appears that males that gain climbing speed with exercise show decreased protein amounts, an unexpected finding. We also found a negative correlation between the change in body mass with exercise and the change in protein amount in females, but only in the analysis of per-weight data (r=−0.50, *P*=0.003). This finding suggests that female animals that lose weight with exercise might experience a shift in body composition towards more protein-rich muscle (lean) mass. Finally, there was no relationship between the change in activity levels experienced by a genotype with exercise and protein amounts (Fig. S7). Together, these analyses suggest that females and males respond differently to exercise, with females exhibiting a shift to more protein when losing weight and males showing increased climbing speed with decreased protein amounts.

To identify the genetic networks that mediate the change in protein levels the animals experience with exercise, we carried out another GWAS, again investigating females, males, combined sexes, and the difference between the sexes. We found 50 genetic variants linked to per-fly change in protein amounts. This number includes nine genetic variants linked to change in protein amount in females, ten in males, and 29 genetic variants identified in the combined sex analysis (two of these also detected in the analysis of female data). Curiously, no genetic variants were linked to the difference between sexes (Table 1). When we analyzed the per-weight change in protein amount, we identified 68 genetic variants. The majority of them were identified for females (30), four were identified for males, four were identified in the combined sex analysis (0 also identified from the male and female data), and seven variants linked to the difference between the sexes. Closer inspection of the QQ plots showed that the female data generally behaves as expected, while from the male data, fewer small *P*-values than expected were detected (Fig. S8). Typically, deviations of *P*-values from the expectation in QQ plots are attributed to population structure. However, the DGRP is an artificial mapping population, and the male and female data are from the same genetic lines, and thus, population structure is not the cause of the deviations here. While other processes might be at play, these results suggest that genetic factors impact the exercise-associated changes in protein amount in females and males differently.

To learn more about the types of genes linked to protein change with exercise, we used GO term analysis. Focusing first on the analysis of per-fly data, we found two clusters of GO terms, one linked to the development and morphogenesis of neurons and response to signals, one linked to Wnt signaling and cell-cell communication (Table S7 and Fig. 4B). The GO terms most enriched among the gene set identified from the per-weight data focused on signal transduction and cell communication (Table S7 and Fig. S6). Comparing the genetic variants linked to exercise-induced changes in protein amounts identified in the analysis of the per-fly data to the variants identified from the per-weight data, we found that there were variants in three genes in common (*Osi17*, *pod1*, *trio*). *Osi17* is involved in cell-cell adhesion, *pod1* is involved in the organization of the cytoskeleton and axon guidance, and *trio* is a signaling molecule (Rho guanine nucleotide exchange factor), illustrating the diverse functions of the genes linked to changes in protein amount in response to exercise (Jenkins et al., 2022; Ozturk-Colak et al., 2024). Together, these analyses reveal that the candidate genes contributing to protein change with exercise have broad roles in development, morphogenesis, and cell-cell communication.

## DISCUSSION

Here, we have used *D. melanogaster* to investigate how exercise impacts body composition, specifically, protein content. We found extensive variation in the protein amounts within the DGRP strain collection, which is influenced strongly by the interaction of sex and genotype, revealing complexities in how protein amounts are controlled. The genetic networks contributing to the body's protein content are similarly complex, with diverse pathways playing a role and the contribution of these pathways differing between the sexes. Thus, our results confirm those of other groups that have measured protein amounts in the DGRP strain collection as part of their studies on metabolomic and transcriptomic variation in these strains (Everett et al., 2020; Zhou et al., 2020): there is significant variation in the protein amounts between strains and between the sexes, which we demonstrated can be modulated by an exercise treatment.

As in other species, the impact of an exercise treatment in *D. melanogaster* is dependent on sex and genotype, with protein amounts showing both increases and decreases (Fig. 1). These findings mimic what we have seen with these animals for body weight change and climbing speed change with exercise. Interestingly, body weight, climbing speed, and protein levels do not change in concert in these animals following an exercise treatment. One might have expected that animals with higher activity levels have higher protein levels and improved climbing speed. However, we see no relationship between the activity levels of the animals and the protein amounts measured here. Furthermore, protein amounts also appear to have no impact on the climbing speed of the animals. This general lack of correlation between measures of activity, climbing speed, and protein amount is likely due to the strong sex-by-genotype interaction effects that impact these traits – and how exercise impacts these traits. Thus, some animals with high protein amount exhibit high activity, while others with similar protein amount show low activity (Fig. S3). It is also possible that the lack of clear relationship between protein levels, activity levels, and climbing speed is due to the use of whole animal protein in this study. In *D. melanogaster*, most muscle is found in the thorax, and thus, using whole animal lysates can obscure changes that occur in muscle in response to exercise, if they are small relative to the whole animal protein levels. The use of whole animal lysates is a limitation of our study, and future studies focused on dissected thorax tissue might provide additional insights.

Several other studies have investigated whole animal protein amount in *D. melanogaster*, but none have done so in the context of exercise treatments. A 2022 study documented that *D. melanogaster* protein amount fluctuated throughout development, ranging from 40-54% of dry matter (Yuan et al., 2022). In 2020, Zhou and colleagues investigated the metabolome of 40 DGRP strains and measured protein amounts as part of the study (Zhou et al., 2020). Similar to our study, Zhou and colleagues found considerable variation in protein amounts in their study population. One of the conclusions drawn by Zhou and colleagues is that in males, body weight is driven by protein content, while in females, body weight is driven mainly by triglyceride content. Thus, they find a correlation between protein amounts and body weight only in males (Zhou et al., 2020). Here, we found that females that show a decrease in body weight with exercise tend to show an increase in protein amount. Taken together with findings from Zhou and colleagues, this observation suggests that females lose weight with exercise due to a change in body composition. This change likely would be a shift from triglycerides to protein, possibly reflecting a switch from fat mass to muscle mass. This hypothesis should be further explored, as it would be of great interest to determine why (a) this response is limited to females and (b) why only some genotypes respond. It is possible that males also show this response to exercise to some degree, but that it is below the level of detection in these studies.

The molecular pathways controlling protein amount in untreated *D. melanogaster* also have been investigated in earlier studies. Everett and colleagues asked how gene expression variation influenced organismal traits in the DGRP, selecting protein amounts as one of these organismal traits (Everett et al., 2020). 55 transcripts had significant associations with protein amounts in males, while 34 transcripts were identified in females (Everett et al., 2020). The transcripts identified in males are linked to neuronal development in GO analysis, a finding that is consistent with the identification of candidate gene linked to neuronal differentiation and morphogenesis in this study. Together, these results suggest that the central nervous system impacts protein amounts. Mechanisms by which this might occur will require further investigation, with one potential mechanism being through the control of feeding and movement behaviors.

Our study suggests that the relationship between protein amount and lifespan might be more complex, as only exercise-treated female animals showed a positive correlation. This relationship was only seen with lifespan data collected in one specific study that matched our study in its design (Ivanov et al., 2015). The variation inherent in the existing lifespan data that we analyzed here – and the low correlations between lifespan seen across studies (Fig. 3B) – highlight the limitation in using lifespan data from previous studies, with different results obtained depending on which lifespan data set was used. While dietary studies across a range of animals have shown that dietary restriction or a protein-restrictive diet can increase lifespan (Kim et al., 2025), little is known about the protein amount of short- versus long-lived animal strains. In a 1996 study, Riha and Luckinbill compared the whole-body protein content of *D. melanogaster* strains selected either for early or delayed reproduction, and thus short- and long-lived (Riha and Luckinbill, 1996). During larval development, the long-lived strains had significantly lower protein amounts, but once the animals reached adulthood, this difference disappeared (Riha and Luckinbill, 1996). If the observation by Riha and Luckinbill carries across diverse genotypes, we would expect that in our study of adult protein amounts, there would be no relationship between protein amount and lifespan. Our findings for the control animals suggest such a lack of association between adult protein amount and lifespan (Fig. 3). However, the findings for exercise-treated animals show some association with lifespan, which suggests that environmental factors such as exercise can modulate the relationship between an animal's protein amount and lifespan. To clarify the relationship between whole-body protein amount and lifespan, studies utilizing dietary restriction that assess protein amounts throughout the animal's lifespan would be helpful, possibly utilizing non-invasive methods such as quantitative magnetic resonance to assay body composition, and it would be important to assay lifespan and protein levels in the same experimental animals.

Our study has some limitations that should be considered in future studies. First, as noted earlier, whole animal protein levels are not an ideal measure of muscle protein levels, as small changes in protein levels in muscle might not be detectable or be masked by changes in other tissues. For example, previous work from our laboratory showed that protein levels in the abdomen and thorax can change in response to exercise, with increase in one, decrease in the other tissue, which likely would result in the detection of no change if whole animals were used (Mendez et al., 2016). Thus, future studies that investigate tissue-specific effects of exercise treatments would provide additional insights into how responses differ between tissues and are then integrated to the whole organism. However, these studies would likely be able to only investigate a small number of genotypes due to the large number of animals and time for dissections required. A second limitation of this study was that we relied on previously published data for two aspects of this study, the correlation analysis with lifespan and the correlation analysis with animal activity. As the analysis in Fig. 3 illustrates, lifespans for the same strains differ significantly between studies, and, ideally, we would have measured lifespan for our animals directly. Due to the small size of *D. melanogaster*, it is not possible to measure protein levels without sacrificing the animal. In future studies, parallel cohorts for the assay of protein levels and lifespan might be an option that can be explored. Finally, while the data about climbing speed and body weight are from the same animals used here to assay protein levels (Watanabe and Riddle, 2021a,b), the data demonstrating the effectiveness of the Treadwheel to increase activity levels and the activity measures for the strains used in this study are from a different cohort of animals, assayed approximately 1 year earlier in our laboratory (Watanabe et al., 2020). In the 2020 study, animals were treated once for 2 h, and we did not assess if the application of the exercise treatment on five consecutive days would change their response. Thus, it is possible that the animals in this study showed a different behavior, with a lower than expected response, which resulted in the lack of correlation between protein levels and activity. To rule out this possibility, future studies would need to be designed to monitor activity levels, protein levels, body weight, and climbing speed on the same cohort of animals.

In summary, the results presented here show that there is a large amount of variation in whole-body protein amounts in *D. melanogaster*, likely at least partially due to variation in muscle mass and body composition. This variation is present in males and females, in control and exercise-treated animals. The animals also vary in how protein amounts change in a response to an exercise treatment, again, due to sex and genotype. While this variation will make it difficult to predict how individuals respond to an exercise treatment – demonstrated for protein amount here, and for other traits in our previous work (Riddle, 2020; Watanabe et al., 2020; Watanabe and Riddle, 2021a,b) – the GWASs presented here identify molecular pathways that can be followed up to gain additional insights. Of particular interest is the link to the central nervous system, as this finding suggests a possible link to behavior.

## MATERIALS AND METHODS

### Fly husbandry

Flies were raised in vials using a standard cornmeal-molasses food supplemented with live yeast (Mendez et al., 2016). Animals were raised in an incubator at 25°C with 70% humidity with a light cycle of 12 h (day/night). All flies used in this study are part of the DGRP strain collection (Huang et al., 2014; Mackay et al., 2012) and are identified by their line number in Table S1.

### Exercise conditions

This experiment utilizes flies from 32 DGRP lines that were collected as part of a previously published experiment investigating the impact of exercise treatment on animal weight and climbing ability (Watanabe and Riddle, 2021a,b) (for an overview, see Fig. 1A). Briefly, virgin animals were collected under standard growth conditions (see previous section), and aged 3-5 days. Half of the animals received a 5-day exercise treatment on the Treadwheel (Mendez et al., 2016) (2 h/day, 10 vials of 10 animals per sex/genotype), while the other half served as unexercised control. On the day following the last exercise treatment, the climbing speed of the animals was measured using a RING assay (Gargano et al., 2005). Afterwards, the flies were weighed in groups of ten and frozen at −80°C for future analyses (Watanabe and Riddle, 2021a,b).

### Fly homogenization

A fly homogenate was prepared to analyze the protein and metabolite concentration of the collected flies (Mendez et al., 2016). 75 μl of lysis buffer (0.01 M KH_2_PO_4_ and 1 mM EDTA), was added to each microfuge tube containing the frozen flies along with two stainless-steel beads. The samples were homogenized using a Benchmark Bead Blaster (Benchmark Scientific, Sayreville, NJ, USA) for one cycle at 5.00 M/s for 6 s. Fly debris was pelleted by centrifugation, the fly homogenate was pipetted into new tube and stored at −80°C for future analyses.

### Protein (Bradford) assay

Protein levels were measured using Bradford reagent [Pierce Coomassie (Bradford) Protein Assay Kit, Thermo Fisher Scientific, Waltham, MA, USA] and a set of standards (bovine serum albumin, 2.0 mg/ml; Fisher Bioreagents, Waltham, MA, USA) (Bradford, 1976). Both the standards and the homogenized fly samples were diluted with lysis buffer (1.5 μl of standard/fly sample and 3.5 μl of lysis buffer). Three technical replicates were assayed using the diluted standards and fly samples (5 μl) and 250 μl of the Bradford reagent. Absorbance was measured using an Epoch microplate spectrophotometer (Agilent BioTek, Santa Clara, CA, USA) at 595 nm, and protein concentrations were calculated based on the standard curve derived from the standards. Protein levels are shown in Table S1. R scripts used to extract the data from the spectrophotometer files, calculate protein levels from the standard curve, and generate summary plots can be found on the Riddle lab GitHub page (https://github.com/riddlenc/Walts_2025).

### Statistical analysis

All statistical analyses were carried out in R v.2023.06.0+421 (Team, 2022). Prior to the analysis, the three technical replicate measurements obtained for each sample were collapsed into a single value for the biological sample by calculating the mean. Samples containing six or fewer flies were removed from the analysis. Generalized linear models were used to evaluate the impact of genotype, sex, and treatment, and the initial model included these three factors as well as their interactions. AIC (Akaike Information Criterion) was used to identify the best model. For the correlation analyses, the two variables of interest for each experimental group were selected, scatterplots were generated, and Pearson's product-moment correlation was calculated (*cor.test* in R). R scripts for all analyses, including the calculation of quantitative genetics parameters (for details see Watanabe and Riddle, 2021b) and correlation analyses, and to generate the figures can be found on the Riddle lab GitHub page (https://github.com/riddlenc/Walts_2025).

### GWAS

Line means for the protein measurements for males and females was used as the input data for a GWAS (Table S2). The GWAS was carried out using the DGRP2 webtool (Huang et al., 2014; Mackay et al., 2012), which implements both a simple regression as well as a mixed effects model. The models control for Wolbachia status as well as five chromosomal inversions that segregate in the DGRP (Huang et al., 2014; Mackay et al., 2012). Based on community standards and inspection of QQ plots, genetic variants with a *P*-value smaller than 10^−5^ were considered as significant.

### GO term analysis

GO analysis was carried out using ShinyGO (0.82) for the genes associated with the genetic variants determined to be significant in the GWAS (Chen et al., 2013; Ge et al., 2020; Kuleshov et al., 2016).

### Lifespan data

Lifespan data for the DGRP strains included in this study was retrieved from the supplemental materials of the following studies: Data from both sexes (Arya et al., 2010; Huang et al., 2020; Ivanov et al., 2015), data from females only (Durham et al., 2014; Jin et al., 2020). The data from Arya et al. (2010) are based on 25 virgin animals of each sex that were housed in five animal groups and checked every 2 days until death (Arya et al., 2010). The data from Ivanov et al. (2015) are a subset of data from Arya et al. (2010) (Arya et al., 2010; Ivanov et al., 2015). The data from Huang et al. (2020) are based on 72 mated animals of each sex, housed in three animal groups, and checked every 2-3 days until death (Huang et al., 2020). The data from Durham et al. (2014) are derived from mated females that were housed individually with a single male present at all times (Durham et al., 2014). At least 22 animals per group were included in the assay, with animals being checked daily (Durham et al., 2014). Data from Jin et al. (2020) are based on 200 mated females housed in groups of 25 (Jin et al., 2020). Deaths were recorded every 2 days, and deaths in the first 10-11 days of the experiment were excluded from the analysis (Jin et al., 2020).

## Supplementary Material

10.1242/biolopen.062342_sup1 Supplementary information

Table S1. Metabolite data. This table includes information about the DGRP strains included in the analysis and all the metabolite data collected. Line – DGRP strain; Treatment – either control (C) or exercise-treated (T); Sex – either male (M) or female (F); Replicate – technical replicate number (1-3); Prot_conc – protein concentration in μg/ml of the sample calculated from the blank-subtracted OD595 measurement based on a standard curve; Vial_weight – weight of the empty epptube in mg; Vial_total – weight of the epptube with the fly sample; Fly_number – number of flies in the sample; Sample_weight – weight of the flies calculated by subtracting the weight of the empty vial (Vial_weight) from the total weight (Vial_total); Prot_conc_weight – protein amount per weight; Prot_conc_number – protein amount per fly.

Table S2. Lines means used for the GWAS analyses.

Table S5. Results from the genome-wide association studies for protein amount per fly. Only genetic variants with a p-value of less than 10^-5^ are shown.

Table S6. Results from the genome-wide association studies for protein amounts per weight. Only genetic variants with a p-value of less than 10^-5^ are shown.

Table S7. GO term analysis results.
