# Supplementary material for: The impact of exercise on protein levels in Drosophila melanogaster
Source: Biol Open. 2026 Jan 29;15(1):bio062342. doi: 10.1242/bio.062342 (PMC12893037; doi:10.1242/bio.062342)
Supplement: Supplementary information [file biolopen-15-062342-s1.pdf]

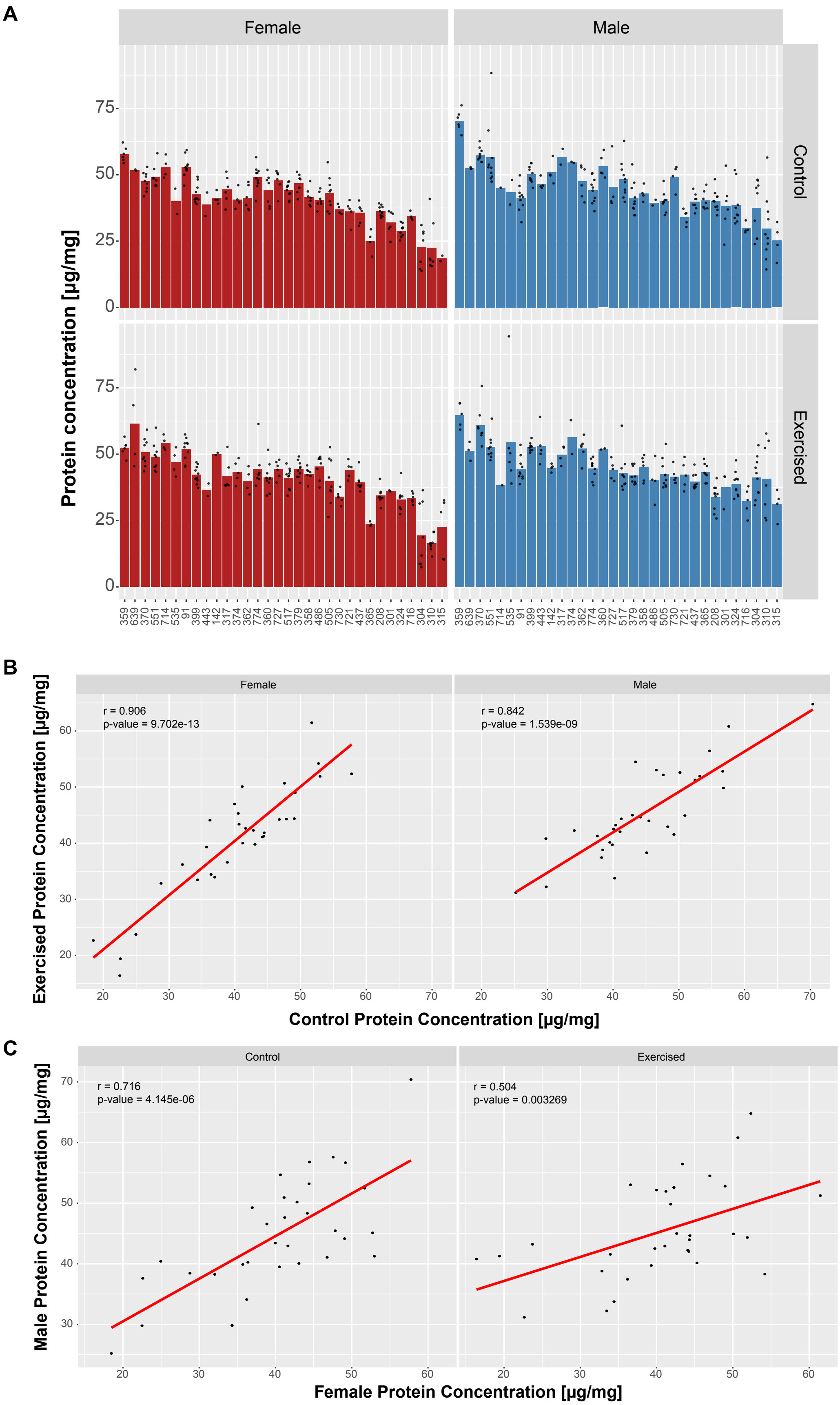

Biology Open • Supplementary information

Fig. S1. Protein amounts per weight are impacted strongly by genotype, both across sex and treatment.

A. Protein amount per weight is shown for control (top) and exercise-treated (bottom) animals separated by sex with females on the left in red and males on the right in blue. X-axis: DGRP line number; Y-axis: protein amount per weight in µg/mg. Group means are shown as bars, with individual data points plotted. n = 1-10 per group, with 10 flies per vial. Each data point represents the mean from 3 technical replicates averaged per vial.

B. Mean protein amount per weight (in µg/mg) of control (X-axis) and exercise-treated (Y-axis) animals are plotted separately against each other for females (left; r = 0.906, p = 9.702e-13) and males (right; r = 0.842, p = 1.539e-09). n = 1–10 biological replicates (vials) per genotype, with 10 flies per vial. P-values and Pearson's correlation coefficient (r) are included within each graph. The trendline is shown in red.

C. Mean protein amount per weight (in µg/mg) of females (X-axis) are plotted against measurements from males (Y-axis) separately for control (left; r = 0.716, p = 4.145e-06) and exercise-treated (right; r = 0.504, p = 0.003269) animals of each DGRP strain. n = 1–10 biological replicates (vials) per genotype, with 10 flies per vial. P-values and Pearson's correlation coefficient (r) are included within each graph. The trendline is shown in red.

Biology Open • Supplementary information

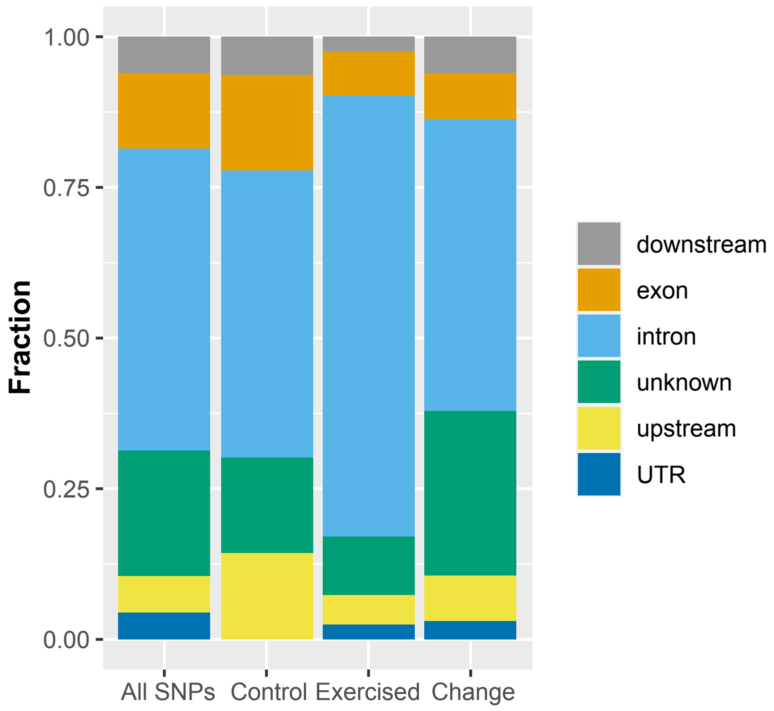

Fig. S2. The genetic variants identified as linked to protein amount per weight are different from the genome-wide distribution for the control samples.

The genetic variants included in the GWAS analysis are classified based on their location relative to genes. The fraction of each class is shown in the stacked bar graph for the total set of variants included in the GWAS (left, “All SNPs”), for the variants identified as significant in the analysis of the protein amount per weight data from control animals (“Control”), and for the variants identified as significant in the analysis of the protein amount per weight data from exercise-treated animals (“Exercised”), as well as for the variants associated with the exercise-induced change in protein levels (“Change”). The distribution is significantly different from the genome-wide pattern only for the analysis of the control samples (chi-square test, p = 0.02599, p = 0.1584, and p = 0.8586).

Biology Open • Supplementary information

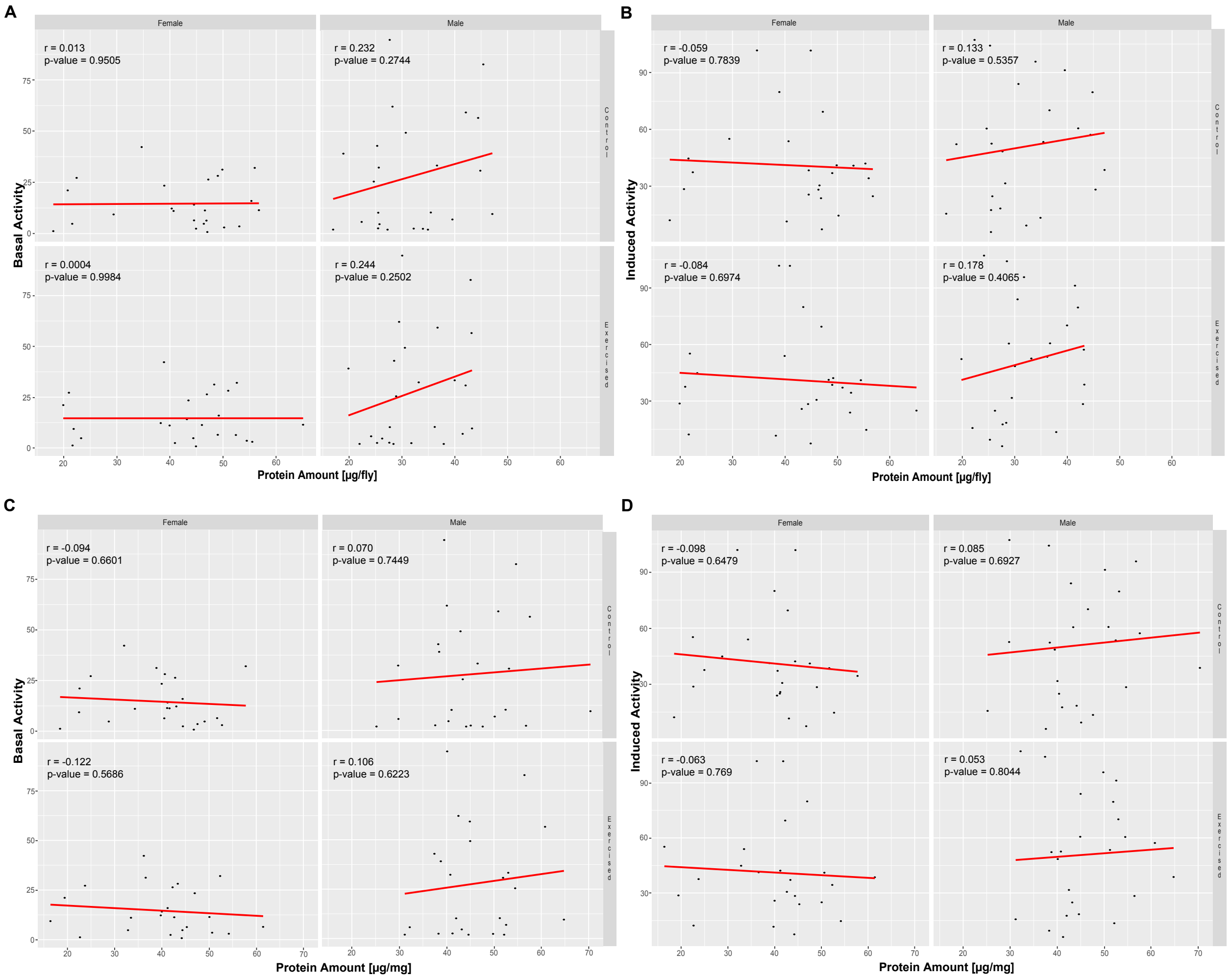

Fig. S3. Protein levels show no correlation with basal or induced activity levels. Activity data are from (Watanabe et al., 2020). Activity levels are the average number of beam crossings recorded by a Drosophila activity monitor in a 5-minute interval over 48 hours. In each graph, p-values and Pearson's correlation coefficient (r) are included, and trendlines are shown in red.

A. Mean protein amount per fly in µg/fly (X-axis) is plotted against basal activity level (Y-axis) and separated by sex and treatment with females (left) and males (right), as well as control (top) and exercise-treated (bottom) for each DGRP line. Correlation between mean protein amounts per fly and basal activity is shown for female-control (r = 0.013; p-value = 0.9505), male-control (r = 0.232; p-value = 0.2744), female-exercised (r = 0.0004; p-value = 0.9984), male-exercised (r = 0.244; p-value = 0.2502).

Biology Open • Supplementary information

B. Mean protein amount per fly in µg/fly (X-axis) is plotted against induced activity level (Y-axis) and separated by sex and treatment with females (left) and males (right), as well as control (top) and exercise-treated (bottom) for each DGRP line. Correlation between mean protein amounts per fly and induced activity is shown for female-control (r = -0.059; p-value = 0.7839), male-control (r = 0.133; p-value = 0.5357), female-exercised (r = -0.084; p-value = 0.6974), male-exercised (r = 0.178; p-value = 0.4065).

C. Mean protein amount per weight in µg/mg (X-axis) is plotted against basal activity level (Y-axis) and separated by sex and treatment with females (left) and males (right), as well as control (top) and exercise-treated (bottom) for each DGRP line. Correlation between mean protein amount per weight and basal activity is shown for female-control (r = -0.094; p-value = 0.6601), male-control (r = 0.070; p-value = 0.7449), female-exercised (r = -0.122; p-value = 0.5686), male-exercised (r = 0.106; p-value = 0.6223).

D. Mean protein amount per weight in µg/mg (X-axis) is plotted against induced activity level (Y-axis) and separated by sex and treatment with females (left) and males (right), as well as control (top) and exercise-treated (bottom) for each DGRP line. Correlation between mean protein amount per weight and induced activity is shown for female-control (r = -0.098; p-value = 0.6479), male-control (r = 0.085; p-value = 0.6927), female-exercised (r = -0.063; p-value = 0.769), male-exercised (r = 0.053; p-value = 0.8044).

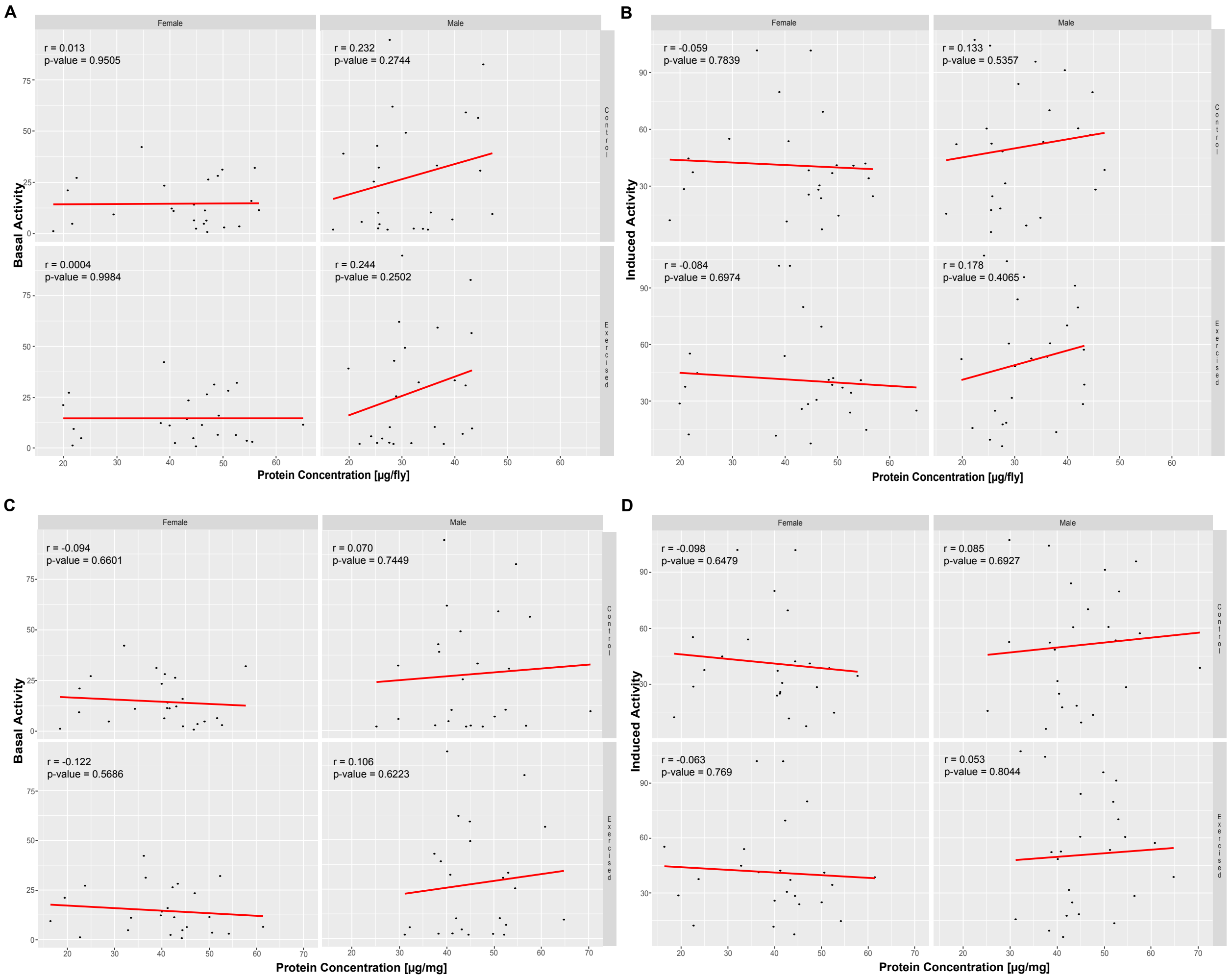

Fig. S4. Climbing speed has a weak negative correlation with protein level. Climbing speed is plotted as a climbing index ranging from 1-4. A value of 4 indicates that all animals in a group reached the top quadrant of a vial in a 2 second time interval, while a value of 1 indicates that all animals remained at the bottom quadrants (see (Watanabe and Riddle, 2021a)for details). p-values and Pearson's correlation coefficients (r) are included within each graph. The trendlines are shown in red.

A. Mean protein amount per fly in µg/fly (X-axis) for each genotype is plotted against climbing speed (Y-axis) and separated by sex (data from females on the left, from males on the right) and treatment (data from control animals in the top panel, from exercise-treated animals in the bottom). Correlation between protein amount per fly and climbing speed is shown for female-control (r = -0.229; p-value = 0.2316), male-control (r = -0.347; p-value = 0.07017), female-exercised (r = -0.181; p-value = 0.3465), male-exercised (r = -0.320; p-value = 0.09095).

Biology Open • Supplementary information

B. Mean protein amount per weight in µg/mg (X-axis) for each genotype are plotted against climbing speed (Y-axis) and separated by sex (data from females on the left, from males on the right) and treatment (data from control animals in the top panel, from exercise-treated animals in the bottom). Correlation between protein amount per weight and climbing speed is shown for female-control (r = -0.295; p-value = 0.1205), male-control (r = -0.377; p-value = 0.04773), female-exercised (r = -0.252; p-value = 0.187), male-exercised (r = -0.310; p-value = 0.1012).

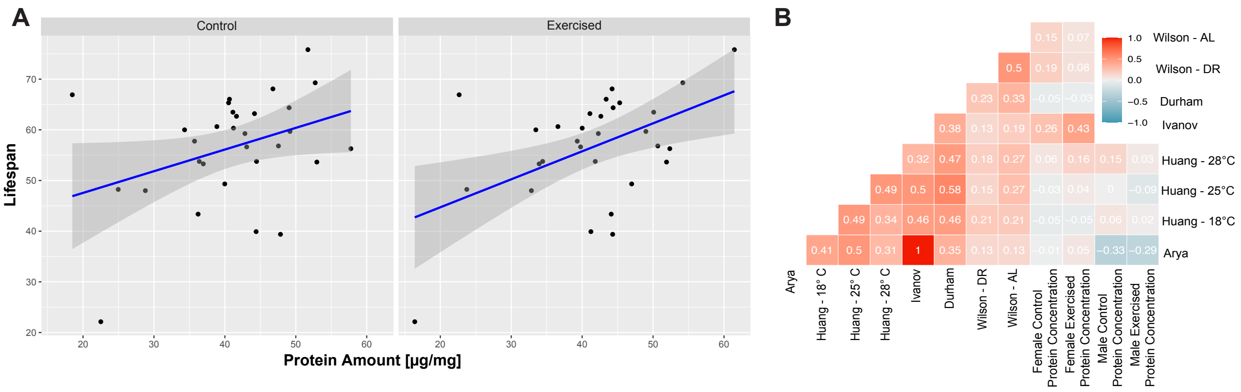

Fig. S5. Life span is strongly correlated to protein concentration in treated animals. A. Lifespan data from females are from (Ivanov et al., 2015). Mean protein amount per weight in µg/mg (X-axis) is plotted against lifespan in days (Y-axis) for control females (left) and exercise-treated females (right) from each genotype. The trendline is shown in blue. **B.** Correlation matrix showing the Pearson correlation coefficients (r; using pairwise complete observations) between lifespan and protein levels (last 4 columns) as well as among the lifespan datasets.

Biology Open • Supplementary information

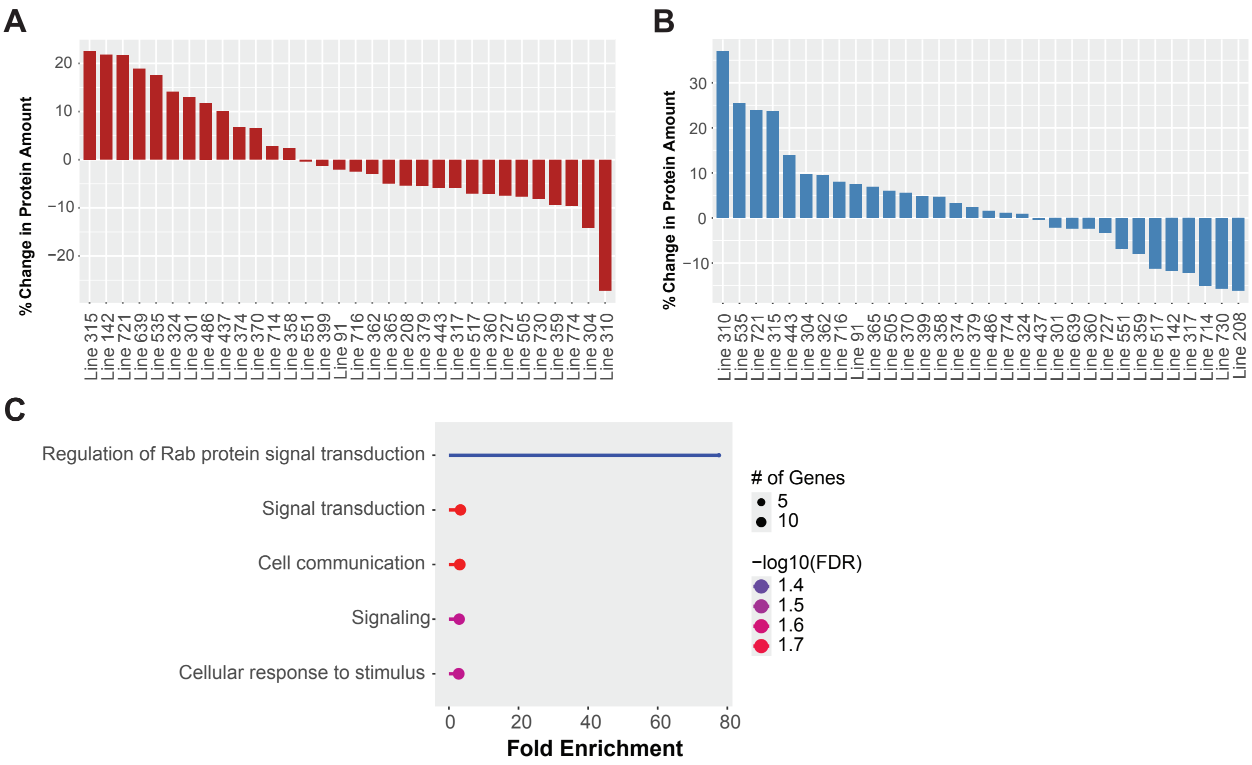

Fig. S6. Genes linked to exercised-induced change in protein level are enriched for functions in signaling.

A. The change in protein amounts per weight with exercise (in percent) is shown for females. X-axis: DGRP line number; Y-axis: change in protein amount per weight in %. n = 1–10 biological replicates (vials) per genotype, with 10 flies per vial.

B. The change in protein amounts per weight with exercise (in percent) is shown for males. X-axis: DGRP line number; Y-axis: change in protein per weight in %. n = 1–10 biological replicates (vials) per genotype, with 10 flies per vial.

C. Biological process GO terms overrepresented among the candidate gene set identified as linked to the exercise-induced change in protein amount per weight by GWAS. The level of overrepresentation is shown as fold enrichment within the graph, while the number of genes within the group is shown by the size of the circle. The color indicates the level of significance for the overrepresentation test.

Biology Open • Supplementary information

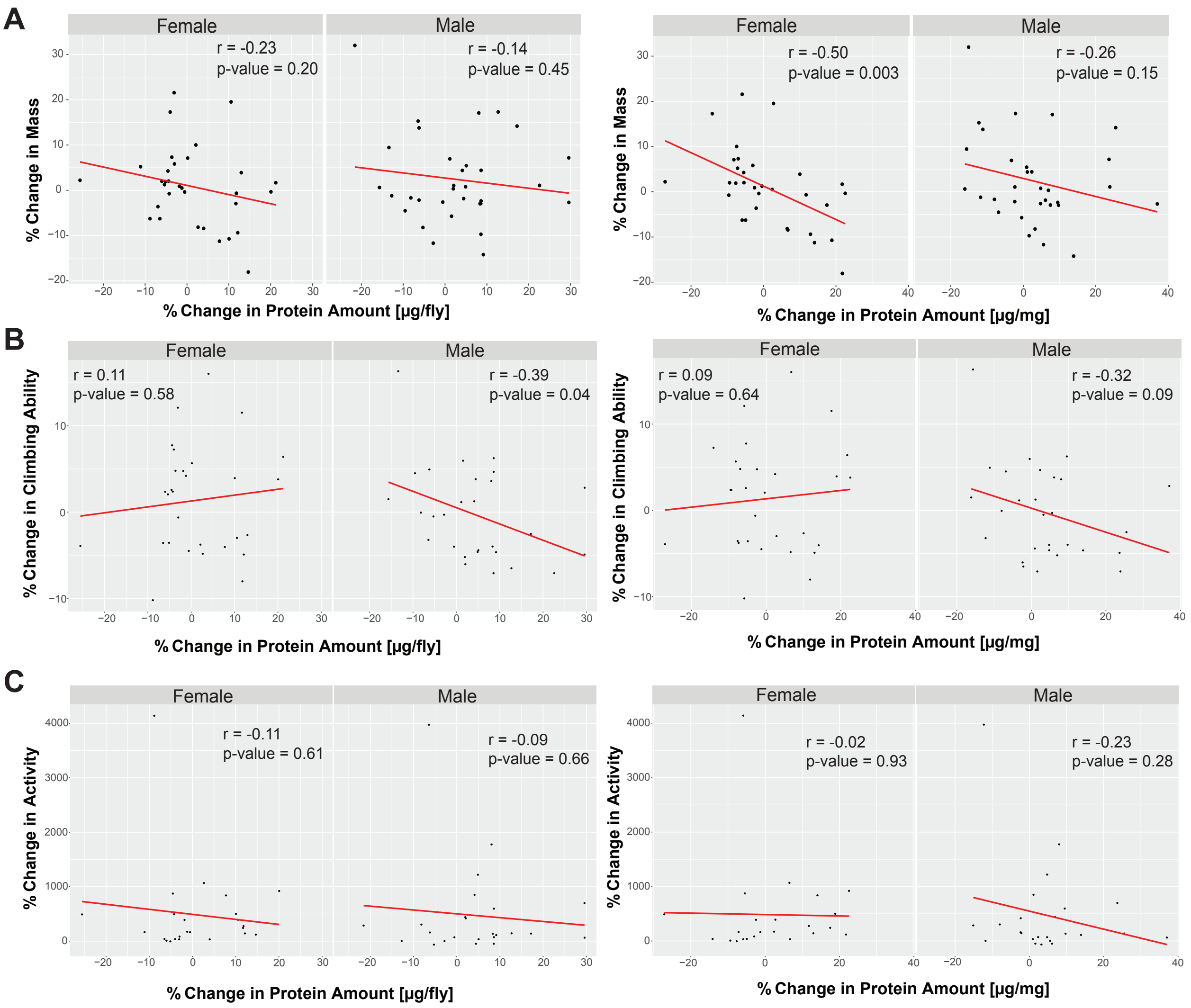

Fig. S7. Exercise-induced change in protein levels is negatively correlated with a change in climbing speed in males. For all plots, the left panel shows protein amounts per fly, while the right panel is based on protein amounts per weight.

A. Percent change in protein levels (X-axis) is plotted against the percent change in mass (Y-axis) for each DGRP line, separately for females and males. Correlation between percent change in protein amount per fly and percent change in mass is shown for females (r = -0.23; p-value = 0.20) and males (r = -0.14; p-value = 0.45). Correlation between percent change in protein amount per weight and percent change in mass is shown for females (r = -0.50; p-value = 0.003) and males (r = -0.26; p-value = 0.15).

B. Percent change in protein levels (X-axis) is plotted against the percent change in climbing speed (Y-axis) for each DGRP line, separately for females and males. Correlation between

percent change in protein amount per fly and percent change in climbing ability is shown for females (r = 0.11; p-value = 0.58) and males (r = -0.39; p-value = 0.04). Correlation between percent change in protein amount per weight and percent change in climbing ability is shown for females (r = 0.09; p-value = 0.64) and males (r = -0.32; p-value = 0.09).

C. Percent change in protein levels (X-axis) is plotted against the percent change in activity levels (Y-axis) for each DGRP line, separately for females and males. Correlation between percent change in protein amount per fly and percent change in activity is shown for females (r = -0.11; p-value = 0.61) and males (r = -0.09; p-value = 0.66). Correlation between percent change in protein amount per weight and percent change in activity is shown for females (r = -0.02; p-value = 0.93) and males (r = -0.23; p-value = 0.28).

Biology Open • Supplementary information

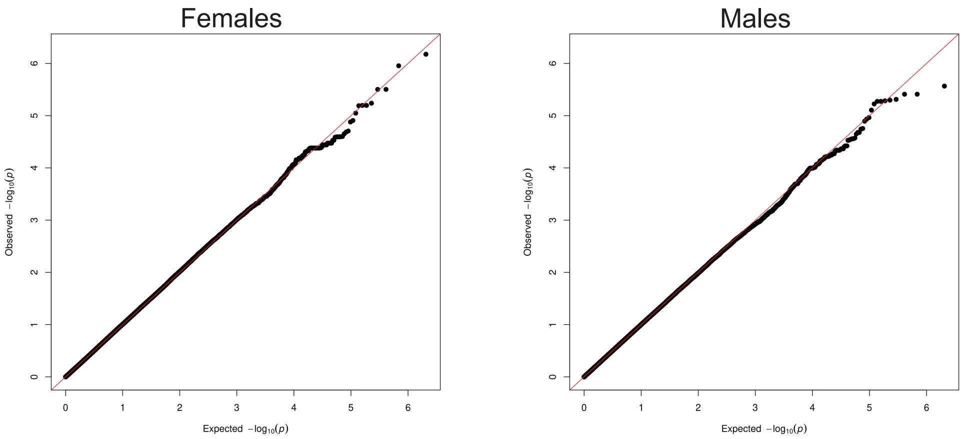

Fig. S8. QQ plots from GWAS investigating the genetic basis of changes in protein level with exercise. These graphs are based on the results from the analysis of protein amounts per fly. Each graph plots expected p-values (X-axis, as $-\log_{10}$ of the p-value) against the observed p-values (Y-axis, as $-\log_{10}$ of the p-value). The red diagonal line shows what the plot would look like if observed and expected values match each other. Data from females are shown on the left, from males on the right.

Table S1. Metabolite data.

This table includes information about the DGRP strains included in the analysis and all the metabolite data collected. Line – DGRP strain; Treatment – either control (C) or exercise-treated (T); Sex – either male (M) or female (F); Replicate – technical replicate number (1-3); Prot_conc – protein concentration in μg/ml of the sample calculated from the blank-subtracted OD595 measurement based on a standard curve; Vial_weight – weight of the empty epptube in mg; Vial_total – weight of the epptube with the fly sample; Fly_number – number of flies in the sample; Sample_weight – weight of the flies calculated by subtracting the weight of the empty vial (Vial_weight) from the total weight (Vial_total); Prot_conc_weight – protein amount per weight; Prot_conc_number – protein amount per fly.

Available for download at
https://journals.biologists.com/bio/article-lookup/doi/10.1242/bio.062342#supplementary-data

Table S2. Lines means used for the GWAS analyses.

Available for download at
https://journals.biologists.com/bio/article-lookup/doi/10.1242/bio.062342#supplementary-data

Biology Open • Supplementary information

Table S3. Generalized linear models identify interactions between line and sex as factor influencing protein levels. Generalized linear models were implemented in R, with the initial model including sex, genotype (Line), treatment, and all interactions. Results from the optimal model based on AIC (Akaike Information Criterion) are shown below. The final model includes sex, genotype, treatment, and a sex-by-genotype interaction. Significance codes: 0 '***' 0.001 '**' 0.01 '*' 0.05 '.' 0.1 ' ' 1

A. Protein amount per fly

	Estimate	Std. Error	t value	Pr(>|t|)
(Intercept)	0.0168067	0.0010662	15.764	< 2e-16 ***
LineLine 208	0.0059172	0.0013477	4.391	1.29e-05 ***
LineLine 301	0.0111858	0.0018646	5.999	3.08e-09 ***
LineLine 304	0.0324243	0.0022371	14.494	< 2e-16 ***
LineLine 310	0.0229271	0.0018700	12.260	< 2e-16 ***
LineLine 315	0.0316640	0.0030304	10.449	< 2e-16 ***
LineLine 317	0.0064959	0.0014880	4.365	1.45e-05 ***
LineLine 324	0.0278305	0.0019088	14.580	< 2e-16 ***
LineLine 358	0.0047998	0.0013889	3.456	0.000579 ***
LineLine 359	0.0016267	0.0013447	1.210	0.226774
LineLine 360	0.0025398	0.0012885	1.971	0.049079 *
LineLine 362	0.0059296	0.0015385	3.854	0.000126 ***
LineLine 365	0.0290254	0.0028849	10.061	< 2e-16 ***
LineLine 370	0.0018030	0.0012613	1.429	0.153285
LineLine 374	0.0032051	0.0014455	2.217	0.026897 *
LineLine 379	0.0050482	0.0013439	3.757	0.000186 ***
LineLine 399	0.0044405	0.0012926	3.435	0.000624 ***
LineLine 437	0.0062690	0.0014295	4.385	1.32e-05 ***
LineLine 443	0.0035700	0.0019017	1.877	0.060874 .
LineLine 486	0.0032724	0.0013322	2.456	0.014262 *
LineLine 505	0.0086462	0.0014116	6.125	1.46e-09 ***
LineLine 517	0.0067537	0.0013398	5.041	5.81e-07 ***
LineLine 535	0.0070323	0.0018453	3.811	0.000150 ***
LineLine 551	0.0025895	0.0012888	2.009	0.044866 *

LineLine 639	0.0042515	0.0017048	2.494	0.012849 *
LineLine 714	0.0021235	0.0014843	1.431	0.152928
LineLine 716	0.0080316	0.0015733	5.105	4.19e-07 ***
LineLine 721	0.0059259	0.0015389	3.851	0.000128 ***
LineLine 727	0.0023980	0.0013661	1.755	0.079610 .
LineLine 730	0.0070046	0.0015785	4.438	1.05e-05 ***
LineLine 774	0.0051785	0.0013313	3.890	0.000109 ***
LineLine 91	0.0007999	0.0012253	0.653	0.514066
TreatmentT	-0.0000273	0.0002793	-0.098	0.922163
SexM	0.0085723	0.0017491	4.901	1.17e-06 ***
LineLine 208:SexM	0.0019606	0.0022518	0.871	0.384215
LineLine 301:SexM	0.0013622	0.0030394	0.448	0.654145
LineLine 304:SexM	-0.0201884	0.0029525	-6.838	1.66e-11 ***
LineLine 310:SexM	-0.0134753	0.0026722	-5.043	5.75e-07 ***
LineLine 315:SexM	-0.0056223	0.0043630	-1.289	0.197923
LineLine 317:SexM	-0.0010954	0.0028150	-0.389	0.697279
LineLine 324:SexM	-0.0016395	0.0029879	-0.549	0.583361
LineLine 358:SexM	0.0024966	0.0025327	0.986	0.324584
LineLine 359:SexM	-0.0049546	0.0021904	-2.262	0.023983 *
LineLine 360:SexM	-0.0052087	0.0023168	-2.248	0.024849 *
LineLine 362:SexM	-0.0038354	0.0024090	-1.592	0.111783
LineLine 365:SexM	-0.0159418	0.0035518	-4.488	8.30e-06 ***
LineLine 370:SexM	-0.0043622	0.0020627	-2.115	0.034776 *
LineLine 374:SexM	-0.0059527	0.0026620	-2.236	0.025629 *
LineLine 379:SexM	0.0072646	0.0023513	3.090	0.002077 **
LineLine 399:SexM	-0.0051307	0.0020809	-2.466	0.013900 *
LineLine 437:SexM	0.0021106	0.0023618	0.894	0.371812
LineLine 443:SexM	-0.0029583	0.0027097	-1.092	0.275307
LineLine 486:SexM	0.0055532	0.0028936	1.919	0.055346 .
LineLine 505:SexM	0.0006412	0.0023317	0.275	0.783390
LineLine 517:SexM	-0.0016705	0.0021991	-0.760	0.447723
LineLine 535:SexM	0.0035344	0.0030324	1.166	0.244175
LineLine 551:SexM	-0.0024802	0.0021001	-1.181	0.237965
LineLine 639:SexM	-0.0017094	0.0028194	-0.606	0.544498

LineLine 714:SexM	0.0072956	0.0043178	1.690	0.091503 .
LineLine 716:SexM	0.0094248	0.0030478	3.092	0.002059 **
LineLine 721:SexM	0.0022077	0.0026437	0.835	0.403938
LineLine 727:SexM	0.0004028	0.0024307	0.166	0.868435
LineLine 730:SexM	-0.0058021	0.0024718	-2.347	0.019168 *
LineLine 774:SexM	0.0053956	0.0022914	2.355	0.018791 *
LineLine 91:SexM	0.0080910	0.0021995	3.679	0.000251 ***

B. Model coefficients for protein amount per weight

	Estimate	Std. Error	t value	Pr(>|t|)
(Intercept)	0.0227038	0.0015127	15.009	< 2e-16 ***
LineLine 208	0.0054992	0.0018585	2.959	0.00318 **
LineLine 301	0.0075596	0.0023074	3.276	0.00110 **
LineLine 304	0.0251975	0.0025193	10.002	< 2e-16 ***
LineLine 310	0.0297935	0.0026192	11.375	< 2e-16 ***
LineLine 315	0.0239589	0.0032475	7.378	4.25e-13 ***
LineLine 317	0.0005233	0.0018639	0.281	0.77898
LineLine 324	0.0099148	0.0019412	5.108	4.14e-07 ***
LineLine 358	0.0010840	0.0018313	0.592	0.55409
LineLine 359	-0.0044873	0.0017362	-2.585	0.00994 **
LineLine 360	0.0008685	0.0017750	0.489	0.62478
LineLine 362	0.0018904	0.0019712	0.959	0.33787
LineLine 365	0.0182697	0.0029416	6.211	8.71e-10 ***
LineLine 370	-0.0022858	0.0017017	-1.343	0.17960
LineLine 374	0.0011511	0.0019470	0.591	0.55457
LineLine 379	-0.0005503	0.0017462	-0.315	0.75274
LineLine 399	0.0008511	0.0017372	0.490	0.62431
LineLine 437	0.0038342	0.0019020	2.016	0.04417 *
LineLine 443	0.0038577	0.0026382	1.462	0.14408
LineLine 486	0.0005731	0.0018001	0.318	0.75030
LineLine 505	0.0014903	0.0017733	0.840	0.40094
LineLine 517	0.0008057	0.0017363	0.464	0.64277
LineLine 535	-0.0002330	0.0021248	-0.110	0.91270
LineLine 551	-0.0022687	0.0017128	-1.325	0.18571

Biology Open • Supplementary information

LineLine 639	-0.0054519	0.0018969	-2.874	0.00416 **
LineLine 714	-0.0039454	0.0018566	-2.125	0.03390 *
LineLine 716	0.0068938	0.0020966	3.288	0.00106 **
LineLine 721	0.0022502	0.0019833	1.135	0.25691
LineLine 727	-0.0009428	0.0018243	-0.517	0.60544
LineLine 730	0.0058152	0.0021075	2.759	0.00593 **
LineLine 774	-0.0013665	0.0017181	-0.795	0.42666
LineLine 91	-0.0035768	0.0016634	-2.150	0.03184 *
TreatmentT	-0.0001092	0.0002645	-0.413	0.67977
SexM	-0.0017808	0.0019318	-0.922	0.35693
LineLine 208:SexM	0.0006613	0.0024241	0.273	0.78509
LineLine 301:SexM	-0.0021151	0.0029707	-0.712	0.47670
LineLine 304:SexM	-0.0207646	0.0029484	-7.043	4.25e-12 ***
LineLine 310:SexM	-0.0215299	0.0031054	-6.933	8.85e-12 ***
LineLine 315:SexM	-0.0093490	0.0040232	-2.324	0.02040 *
LineLine 317:SexM	-0.0021960	0.0025601	-0.858	0.39129
LineLine 324:SexM	-0.0048866	0.0024814	-1.969	0.04928 *
LineLine 358:SexM	0.0006977	0.0024833	0.281	0.77881
LineLine 359:SexM	-0.0016454	0.0022341	-0.736	0.46167
LineLine 360:SexM	-0.0028322	0.0024442	-1.159	0.24693
LineLine 362:SexM	-0.0027169	0.0024954	-1.089	0.27659
LineLine 365:SexM	-0.0152678	0.0033337	-4.580	5.44e-06 ***
LineLine 370:SexM	-0.0016656	0.0021897	-0.761	0.44709
LineLine 374:SexM	-0.0040214	0.0027197	-1.479	0.13966
LineLine 379:SexM	0.0037655	0.0023050	1.634	0.10275
LineLine 399:SexM	-0.0022555	0.0022297	-1.012	0.31207
LineLine 437:SexM	0.0004296	0.0024608	0.175	0.86144
LineLine 443:SexM	-0.0047806	0.0030960	-1.544	0.12298
LineLine 486:SexM	0.0036256	0.0027335	1.326	0.18513
LineLine 505:SexM	0.0018292	0.0023267	0.786	0.43200
LineLine 517:SexM	0.0002467	0.0022593	0.109	0.91309
LineLine 535:SexM	-0.0012741	0.0026835	-0.475	0.63509
LineLine 551:SexM	-0.0003661	0.0022023	-0.166	0.86802
LineLine 639:SexM	0.0038671	0.0025881	1.494	0.13554

LineLine 714:SexM	0.0070561	0.0035430	1.992	0.04678 *
LineLine 716:SexM	0.0043382	0.0029819	1.455	0.14613
LineLine 721:SexM	0.0030755	0.0026852	1.145	0.25242
LineLine 727:SexM	0.0024878	0.0025024	0.994	0.32046
LineLine 730:SexM	-0.0042689	0.0026879	-1.588	0.11266
LineLine 774:SexM	0.0030263	0.0022533	1.343	0.17966
LineLine 91:SexM	0.0060806	0.0022234	2.735	0.00639 **

Biology Open • Supplementary information

Table S4. Quantitative genetics parameters. Various quantitative genetics parameters such as heritability are reported for protein amounts per fly and per weight in the control and exercise-treated animals.

Parameter	Symbol	Protein amount (per fly)		Protein amount (per weight)	
		Control	Treated	Control	Treated
Mean	μ	37.779	37.545	42.585	42.683
Genetic variance	σ_G^2	84.244	76.714	80.997	74.503
Genetic standard deviation	σ_G	9.178	8.759	9.000	8.632
Environmental variance	σ_E^2	22.290	20.640	31.187	36.192
Environmental standard deviation	σ_E	4.721	4.5432	5.585	6.016
Phenotypic variance	σ_P^2	106.534	97.354	112.184	110.696
Phenotypic standard deviation	σ_P	10.322	9.867	10.592	10.521
Heritability	H_2	0.791	0.788	0.722	0.673
Coefficient of genetic variation	CV_G	24.295	23.328	21.134	20.222
Coefficient of environmental variation	CV_E	12.497	12.100	13.114	14.094
Cross-sex genetic correlation	r_{MF}	0.719	0.521	0.764	0.567
Genetic correlation	r_g	0.751	0.609	0.762	0.588

Biology Open • Supplementary information

Table S5. Results from the genome-wide association studies for protein amount per fly. Only genetic variants with a p-value of less than 10^{-5} are shown.

Available for download at
https://journals.biologists.com/bio/article-lookup/doi/10.1242/bio.062342#supplementary-data

Table S6. Results from the genome-wide association studies for protein amounts per weight. Only genetic variants with a p-value of less than 10^{-5} are shown.

Available for download at
https://journals.biologists.com/bio/article-lookup/doi/10.1242/bio.062342#supplementary-data

Table S7. GO term analysis results.

Available for download at
https://journals.biologists.com/bio/article-lookup/doi/10.1242/bio.062342#supplementary-data

Table S8. Correlations between protein levels and other exercise-related traits. Correlation analysis from protein amounts per weight, showing Pearson's correlation coefficients as well as p-values).

Sex	Phenotype 1	Phenotype 2	Correlation coefficient (r^2) (p-value)
Female	Protein concentration - control	Activity levels - basal	-0.095 (0.6601)
Male	Protein concentration - control	Activity levels - basal	0.070 (0.7449)
Female	Protein concentration – exercise-treated	Activity levels - basal	-0.122 (0.5686)
Male	Protein concentration – exercise-treated	Activity levels - basal	0.106 (0.6223)
Female	Protein concentration - control	Activity levels – exercise-induced	-0.098 (0.6479)
Male	Protein concentration - control	Activity levels - exercise-induced	0.085 (0.6927)
Female	Protein concentration – exercise-treated	Activity levels - exercise-induced	-0.063 (0.769)
Male	Protein concentration – exercise-treated	Activity levels - exercise-induced	0.053 (0.8044)
Female	Protein concentration - control	Climbing speed - control	-0.295 (0.1205)
Male	Protein concentration - control	Climbing speed - control	-0.377 (0.04773)
Female	Protein concentration – exercise-treated	Climbing speed – exercise-treated	-0.252 (0.187)
Male	Protein concentration – exercise-treated	Climbing speed – exercise-treated	-0.310 (0.1012)

Biology Open • Supplementary information
